# Germline variants in the *SEMA4A* gene predispose to familial colorectal cancer type X

**DOI:** 10.1038/ncomms6191

**Published:** 2014-10-13

**Authors:** Eduard Schulz, Petra Klampfl, Stefanie Holzapfel, Andreas R. Janecke, Peter Ulz, Wilfried Renner, Karl Kashofer, Satoshi Nojima, Anita Leitner, Armin Zebisch, Albert Wölfler, Sybille Hofer, Armin Gerger, Sigurd Lax, Christine Beham-Schmid, Verena Steinke, Ellen Heitzer, Jochen B. Geigl, Christian Windpassinger, Gerald Hoefler, Michael R. Speicher, C. Richard Boland, Atsushi Kumanogoh, Heinz Sill

**Affiliations:** 1 Division of Hematology, Department of Internal Medicine, Medical University of Graz, A-8036 Graz, Austria; 2 Department of Internal Medicine, General Hospital Graz West, A-8020 Graz, Austria; 3 Institute of Human Genetics, Faculty of Medicine, University of Bonn, D-53127 Bonn, Germany; 4 Division of Human Genetics, Department of Medical Genetics, Molecular and Clinical Pharmacology, Medical University of Innsbruck, A-6020 Innsbruck, Austria; 5 Department of Pediatrics I, Medical University of Innsbruck, A-6020 Innsbruck, Austria; 6 Institute of Human Genetics, Medical University of Graz, A-8036 Graz, Austria; 7 Clinical Institute of Medical and Chemical Laboratory Diagnostics, Medical University of Graz, A-8036 Graz, Austria; 8 Institute of Pathology, Medical University of Graz, A-8036 Graz, Austria; 9 Department of Immunopathology, WPI Immunology Frontier Research Center, Osaka University, Suita City, Osaka 565-0871, Japan; 10 Department of Pathology, Osaka University Graduate School of Medicine, Osaka University, Suita City, Osaka 565-0871, Japan; 11 Department of Ear, Nose and Throat, Medical University of Graz, A-8036 Graz, Austria; 12 Division of Oncology, Department of Internal Medicine, Medical University of Graz, A-8036 Graz, Austria; 13 Department of Pathology, General Hospital Graz West, 8020 Graz, Austria; 14 Division of Gastroenterology, Baylor University Medical Center, Dallas, Texas 75246-2017, USA; 15 Department of Respiratory Medicine, Allergy and Rheumatic Disease, Graduate School of Medicine, Osaka University, Suita City, Osaka 565-0871, Japan; 16 JST, CREST, Suita City, Osaka 565-0871, Japan

## Abstract

Familial colorectal cancer type X (FCCTX) is characterized by clinical features of hereditary non-polyposis colorectal cancer with a yet undefined genetic background. Here we identify the *SEMA4A* p.Val78Met germline mutation in an Austrian kindred with FCCTX, using an integrative genomics strategy. Compared with wild-type protein, SEMA4A^V78M^ demonstrates significantly increased MAPK/Erk and PI3K/Akt signalling as well as cell cycle progression of SEMA4A-deficient HCT-116 colorectal cancer cells. In a cohort of 53 patients with FCCTX, we depict two further *SEMA4A* mutations, p.Gly484Ala and p.Ser326Phe and the single-nucleotide polymorphism (SNP) p.Pro682Ser. This SNP is highly associated with the FCCTX phenotype exhibiting increased risk for colorectal cancer (OR 6.79, 95% CI 2.63 to 17.52). Our study shows previously unidentified germline variants in *SEMA4A* predisposing to FCCTX, which has implications for surveillance strategies of patients and their families.

## Abstract (web summary)

It is known that hereditary factors contribute to familial colorectal cancer type X. Here, the authors uncover the SEMA4A p.Val78Met germline mutation and show that the protein product is associated with changes in cell cycle progression in colorectal cancer cells.

## Graphical abstract

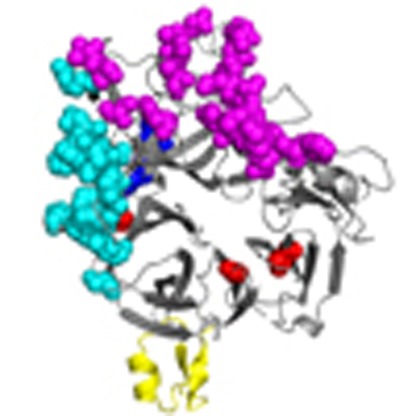

Colorectal cancer (CRC) is the third most common cancer worldwide^1^. Approximately 5% of cases are inherited in an autosomal dominant manner with familial adenomatous polyposis and hereditary non-polyposis colorectal cancer (HNPCC) being the two major hereditary forms^2^,^3^. HNPCC is clinically diagnosed when Amsterdam-I or -II criteria (AC-I/-II) are met: three or more relatives affected through at least two generations by CRC (AC-I) or an HNPCC-associated cancer (AC-II), respectively, with one patient being a first-degree relative of the other two and one diagnosed before the age of 50 years^4^. However, 40 to 50% of patients with HNPCC fulfilling AC-I lack detectable germline mutations in cancer predisposition genes and are classified as familial colorectal cancer type X (FCCTX)^5^,^6^,^7^. In contrast to Lynch syndrome (LS)—the HNPCC entity characterized by germline DNA mismatch repair (MMR) gene mutations and somatically acquired microsatellite instability—individuals with FCCTX exhibit decreased risk for extracolonic neoplasms, that is, endometrial, stomach, small bowel and urinary tract carcinomas and tumour formation including CRC development tends to occur at a later age^5^,^8^,^9^. It is expected that single uncommon susceptibility genes transmitted in an autosomal dominant manner are responsible for a subset of FCCTX cases, which in turn implies that this syndrome is likely to be heterogeneous^2^,^5^,^8^. Here we show that germline variants in the semaphorine 4A (*SEMA4A*) gene confer susceptibility to FCCTX. This finding broadens our understanding of the biology of those malignancies and forms the basis for effective cancer detection and prevention strategies.

## Results

### Pedigree analysis and variant identification

**Figure 1:**
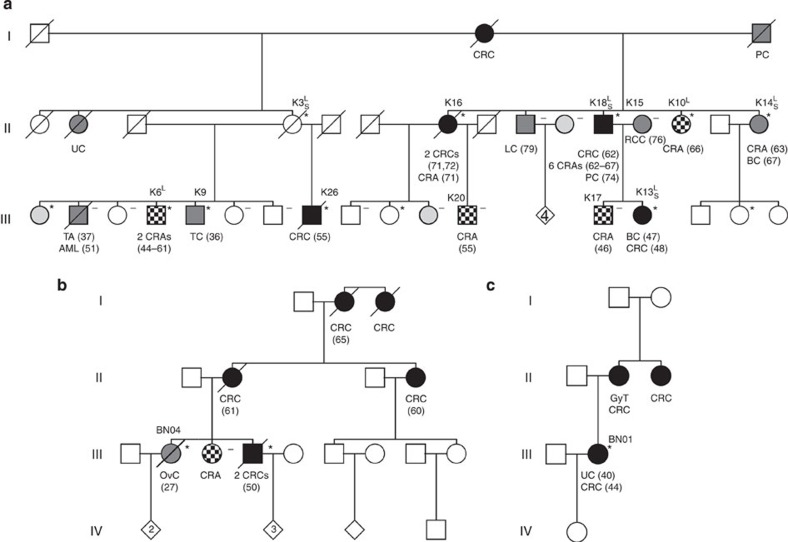
Pedigrees of families with germline *SEMA4A* mutations. Families with V78M (**a**), G484A (**b**) and S326F (**c**) mutations are shown. L, individual included in LA; S, individual included in WES; asterisk, *SEMA4A* mutation carrier; minus, *SEMA4A* wild type; black symbol, CRC; checkered symbol, colorectal adenoma; dark grey, malignant neoplasm; light grey, benign neoplasm; number in symbol, number of unspecified offspring. AML, acute myeloid leukaemia; BC, breast cancer; CRA, colorectal adenoma; GyT, gynaecologic tumour; OvC, ovarian cancer; PC, prostate cancer; TA, thyroid adenoma; TC, testicular cancer; UC; uterine cancer; UT, uterine tumour. Results of mutational analyses are indicated in tested individuals only. Age at diagnosis (years) is given in parentheses. For multiple colorectal adenomas, age at first presentation or at screening colonoscopy is indicated. An extended pedigree of the family with the V78M mutation including age of the individuals is shown in Supplementary Fig. 1, histopathological characteristics of their colorectal neoplasms are summarized in Table 1.

In the course of a previous study focusing on pedigree analysis of patients with therapy-related myeloid neoplasms^10^,^11^, we have identified a large Austrian kindred with FCCTX (Family K, Fig. 1a; Supplementary Fig. 1). CRCs in this family were inherited in an autosomal dominant pattern with incomplete penetrance meeting AC-I. In each affected individual, one to six colorectal adenomas and one to two CRCs were diagnosed at a median age of 62.5 years (range, 44–72). The majority of colorectal neoplasms was located in the distal colon and rectum and showed tubular histological features without evidence for an increase of infiltrating lymphocytes (Table 1).

We conducted genetic linkage analysis (LA) of five family members with colorectal neoplasms and one unaffected, putative mutation carrier (Fig. 1a), which revealed four shared regions on chromosomes 1, 3, 10 and 20 (Supplementary Fig. 2), none of them harbouring known cancer-associated genes. We next performed whole-exome sequencing (WES) on four of these individuals (Fig. 1a). A heterozygous germline variant was identified in the *MUTYH* gene (NM_001128425.1:c.650G>A:p.Arg217His, rs147754007) in the first-degree relatives K13 and K18 but not in individuals K3 and K14 (Supplementary Fig. 3). We, therefore, excluded *MUTYH* R217H as a culprit germline mutation responsible for the majority of neoplasms in this family, which is in line with the fact that *MUTYH*-associated polyposis is an autosomal recessive CRC predisposition syndrome^12^. To identify novel candidate causative mutations, we combined LA and WES and filtered heterozygous, non-synonymous protein-coding or splice-site variants with a minor allele frequency of ≤0.01 (Supplementary Table 1). All variants were confirmed by Sanger sequencing and analysed in two further family members with CRC (K16 and K26). Only variant p.Val78Met (NM_001193300:c.232G>A) in the *SEMA4A* gene located on chromosome 1q22 was shared by all tested individuals. However, in this approach, we included two individuals with colorectal adenomas constituting a frequent but not obligate part of HNPCC syndromes^13^. As this might constitute a potential bias, we focused in an independent analysis on variants from WES shared by individuals with CRC (K13, K18) or with an offspring with CRC (K3). Of 24 variants identified (Supplementary Table 2), two were also present in individuals K16 and K26. We excluded the p.Val212Phe variant in *ZNF763* (rs7249379) due to non-conservation because Phe212 represents the common chimpanzee allele. Only *SEMA4A* V78M segregated with all CRC cases and was also detected in individuals K9 with testicular and K14 with breast cancer, respectively (Fig. 1a). Given a mean age of 61 years of individuals with FCCTX at disease onset^5^, we estimated a phenocopy rate of 0.00 and a penetrance rate of 0.56 of the *SEMA4A* V78M variant in Family K. cDNA from peripheral blood (PB) leukocytes demonstrated expression of the mutant allele (Supplementary Fig. 4).

**Figure 2:**
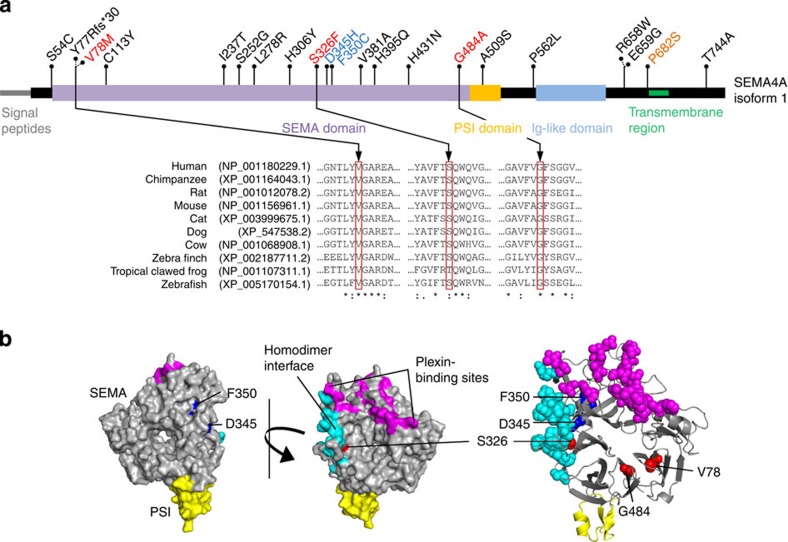
Localization of germline and somatic CRC *SEMA4A* mutations at the protein level. (**a**) Germline mutations found in this study are illustrated in red, the SNP in orange, germline mutations associated with eye diseases in blue and somatic CRC mutations in black, respectively. Multiple sequence alignments of SEMA4As of selected species are shown below. Note that class 4 semaphorins can only be found in vertebrates. (**b**) SEMA and PSI domains (55–527, yellow) of human SEMA4A were modelled primarily to SEMA4D (1OLZ). Eye disease-associated residues D345 and F350 are located in the back of the protein below the plexin binding sites (magenta). V78 and G484 have no contact to the surface, are spatially distinct from D345 and F350 but are located in juxtaposition in β-propellers 1 and 7, respectively. S326 is part of the homodimer interface (cyan) having surface contact.

SEMA4A is a membrane-bound class 4 semaphorin receptor with organ-specific and immunomodulatory effects as well as growth regulatory functions^14^,^15^,^16^. V78M lies within the SEMA domain responsible for receptor binding and Val78 is well conserved (Fig. 2a; Supplementary Fig. 5). This variant is absent from dbSNP137, the 1000 Genomes Project database and the National Heart, Lung and Blood Institute Exome Variant Server (ESP6500). Prediction tools favour consequences for its protein function (SIFT score=0, PolyPhen-2 score=0.987, vertebrate Phylop100 score=7.434, vertebrate PhastCons100 score=1, phastConsElements100 score=407 [LOD=65] and MutationTaster 2=disease causing with 0.95 probability value).

### Recurrent somatic mutations in CRCs of *SEMA4A* V78M carriers

**Figure 3:**
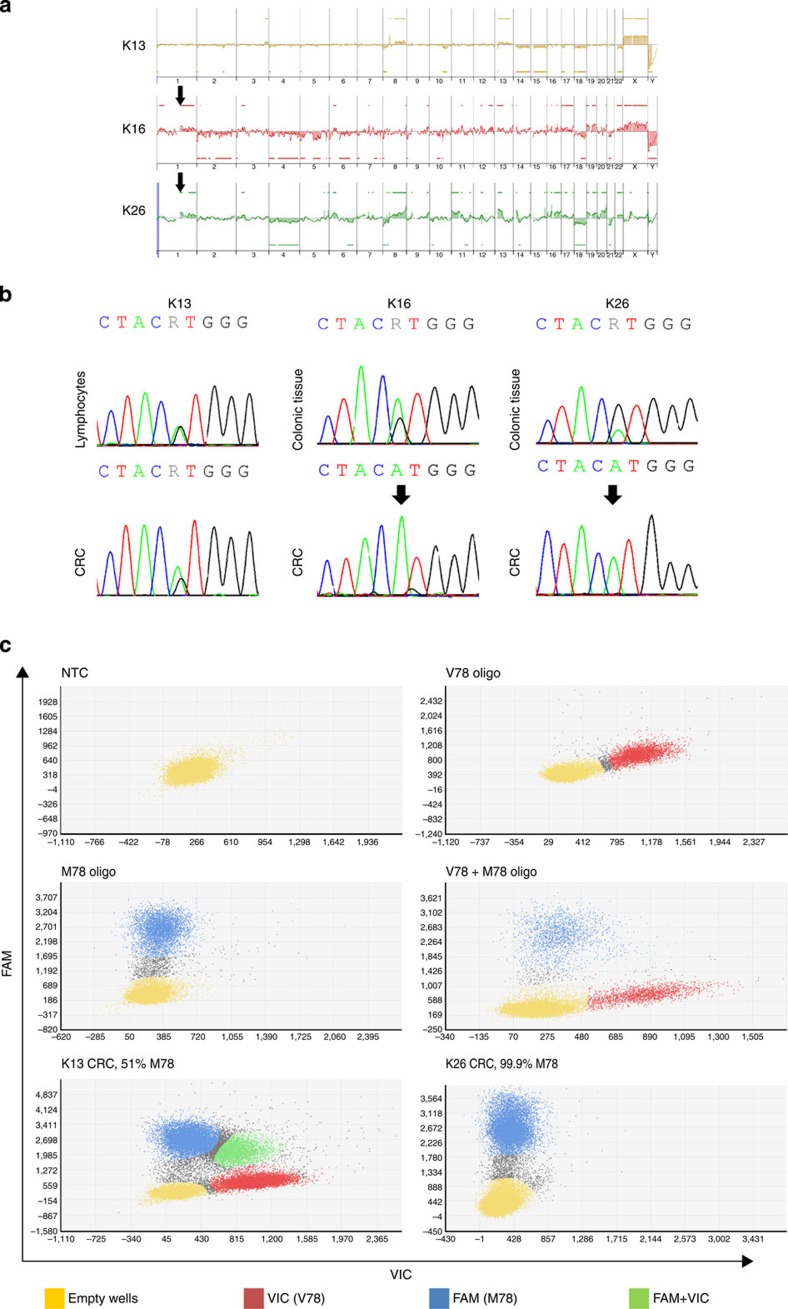
LOH in CRCs of patients K16 and K26. (**a**) Array-based comparative genomic hybridization of three CRCs from Family K with germline *SEMA4A* V78M mutation. A gain in the *SEMA4A* locus is marked with an arrow. (**b**) Sanger sequencing. (**c**) Quantitative dPCR using fluorophore-coupled (VIC, FAM) TaqMan probes specific for wild-type (V78) or mutant (M78) *SEMA4A* nucleotide variants. Each dot represents a single well on a 20K chip. The performance of this assay was tested with specific oligonucleotide templates. The confidence level was set to 95% and the desired precision value was 10%. NTC, no template control.

We then analysed CRC specimens of mutation carriers for copy-number alterations by array-based comparative genomic hybridization and loss of heterozygosity (LOH) by Sanger sequencing, respectively. Gains on the long arm of chromosome 1 involving the *SEMA4A* locus were observed in two of three CRCs together with a homozygous *SEMA4A* V78M status (Fig. 3). We did not detect copy-number alterations in the *MUTYH* gene in any of the three analysed CRCs including the heterozygous R217H carrier K13. We also analysed four available CRCs for recurrent, somatically acquired mutations in known CRC genes by targeted deep sequencing and identified mutations in *TP53* in 3/4, *APC* in 2/4, *KRAS* in 2/4 and *PIK3CA* in 1/4 CRC cases, respectively, as possible cooperating events (Table 2). Notably, there was no predominance of C:G to A:T transversion mutations in the CRC of patient K13 characteristic for complete loss of MUTYH activity^12^.

### SEMA4A^V78M^ affects proliferative pathways

**Figure 4:**
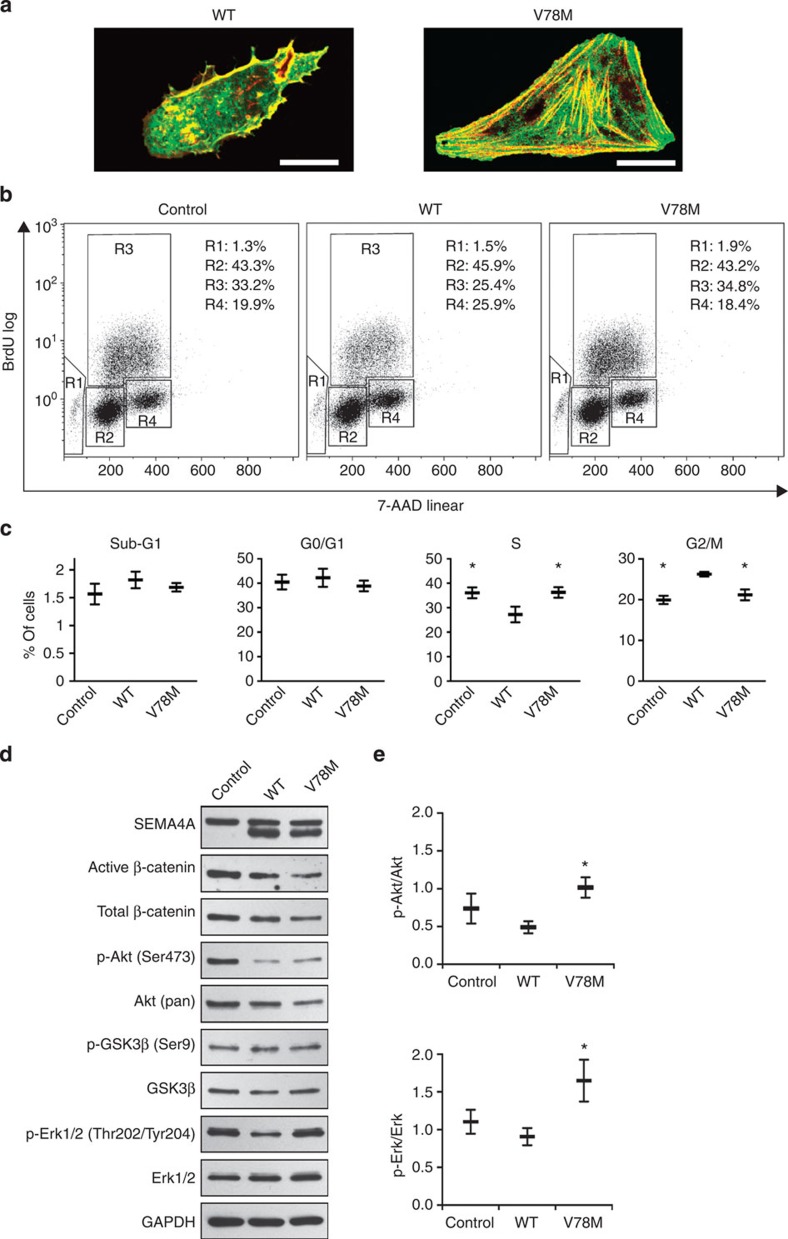
SEMA4A^V78M^ shows normal surface expression and leads to cell cycle changes in HCT-116 cells. (**a**) ARPE-19 cells were transfected with the plasmid constructs expressing Sema4A^WT^-EGFP or Sema4A^V78M^-EGFP proteins, incubated for 48 h and stained with phalloidin. Green, Sema4A-EGFP; red, phalloidin (actin). Representative images obtained by confocal microscopy are shown. The size of the scale bar is 20 μm. (**b**,**c**) Representative density plots and statistical analysis of GFP-positive SEMA4A-transfected HCT-116 cells stained by 7-AAD and APC anti-BrdU antibodies for cell cycle analysis. Cells were analysed 48 h after transfection. Significantly, more SEMA4A^V78M^ than SEMA4A^WT^-transfected cells are in S phase and significantly less in G2/M phase, respectively (mean±s.e.m.; *n*=3 per group; two-tailed paired Student's *t*-test; *P<0.05 compared with WT). Cell cycle phase: Sub-G1 (R1), G1/G0 (R2), S (R3), G2/M (R4). (**d**,**e**) Representative immunoblots and statistical analysis of SEMA4A-transfected HCT-116 cells (whole-cell lysates) lyzed 48 h after transfection. SEMA4A^V78M^-transfected cells show increased phosphorylation of Akt and Erk (mean±s.e.m.; *n*=6 per group; two-tailed paired Student's *t*-test; *P<0.05 compared with WT). (p-)GSK3β and (active) β-catenin proteins were blotted on a separate membrane in this experiment. No effects on GSK3β and β-catenin phosphorylation were seen in repeated experiments.

Compound heterozygous germline mutations in *SEMA4A* have been reported in patients with retinal degenerative diseases and studies in knock-in mice showed that one of these mutations (F350C) leads to an abnormal Sema4A localization in retinal pigment epithelial cells^17^,^18^. A three-dimensional protein model of human SEMA4A predicts that Val78 has no spatial relationship to residues associated with retinal disorders (Fig. 2b). In agreement with this prediction and the family's history lacking apparent ocular manifestations, the expression of a fusion gene composed of Sema4A^V78M^ and carboxyl-terminal green fluorescent protein (GFP) in human retinal ARPE-19 cells showed normal GFP signal distribution (Fig. 4a).

*SEMA4A* is widely expressed including normal colonic tissue (Supplementary Fig. 6) but is undetectable in 2/4 CRC cell lines analysed (Supplementary Fig. 7). It has been shown to have inhibitory effects on proliferation and migration of endothelial cells by antagonizing vascular endothelial growth factor^16^. We therefore analysed transiently transfected SEMA4A-deficient HCT-116 cells characterized by *KRAS* and *PIK3CA* mutations. We were unable to demonstrate significant differences between wild-type and mutant SEMA4A on migration (Supplementary Fig. 8). However, as compared with SEMA4A^wt^, significantly more SEMA4A^V78M^-transfected cells were in S phase under normal growth conditions (Fig. 4b,c). We then assessed activation of the phosphoinositide 3-kinase/Akt (PI3K/Akt), mitogen-activated protein kinase/extracellular signal-regulated kinase (MAPK/Erk) and Wnt/β-catenin pathways that have been shown to be important in colorectal carcinogenesis^19^. As compared with SEMA4A^wt^, SEMA4A^V78M^-transfected HCT-116 cells revealed significantly enhanced activation of the PI3K/Akt and MAPK/Erk pathways both mediating proliferation by increasing cells in S phase and accelerating G2/M transition (Fig. 4d,e; Supplementary Fig. 9)^20^,^21^,^22^. Transient transfection of 293T cells, however, showed no effect of SEMA4A on the PI3K/Akt pathway (Supplementary Fig. 10).

### *SEMA4A* variants are associated with FCCTX

To study the prevalence of *SEMA4A* germline mutations in FCCTX, we screened 53 unrelated FCCTX cases from Austria, Germany and the United States (Supplementary Table 3) and identified two further mutations located in the SEMA domain (heterozygous c.1451G4C, p.Gly484Ala, rs148744804; homozygous c.977C4T, p.Ser326Phe; Supplementary Fig. 11). These mutations affect highly conserved residues (Fig. 2a and Supplementary Figs 12 and 13) and prediction tools indicate an effect on protein function for both of them (Supplementary Table 4). The G484A variant has a global minor allele frequency of 0.001 in the 1000 Genomes Project and ESP6500 databases. It was also found in the index patient's brother affected with CRC (Fig. 1b; Supplementary Fig. 11). The novel S326F variant affects a residue predicted to be involved in homodimer formation (Fig. 2b; Supplementary Fig. 12). Furthermore, we detected the heterozygous single-nucleotide polymorphism (SNP) p.Pro682Ser (c.2044C>T, rs76381440) in six of 47 (13%) German and Austrian FCCTX patients, respectively (Supplementary Table 3; Supplementary Fig. 11). We, therefore, initiated a genetic association study using DNA from 1,138 Caucasian control subjects from Austria without a personal or family history of cancer. These specimens were collected previously during the course of a local health screening study^23^. The P682S SNP demonstrated a highly significant association with the FCCTX phenotype resulting in an increased risk for CRC (Table 3). Screening the 1000 Genomes Project data base revealed a comparable prevalence of heterozygotes among European individuals of 2.0%.

### *SEMA4A* is somatically mutated in sporadic cancers

Finally, we were interested whether somatically acquired *SEMA4A* mutations are prevalent in sporadic CRCs as well as other neoplasms. Analysis of confirmed mutations across different cancer types revealed that *SEMA4A* mutations occur in 2.7% (15/559) of colorectal, 2.8% (6/212) of stomach and 3.3% (8/241) of uterine cancers^24^,^25^. In 92% of them, they constitute missense mutations (Supplementary Table 5) scattered throughout the gene (Fig. 2a). Data from the cBioPortal for Cancer Genomics indicate that the *SEMA4A* gene is amplified in a wide range of different tumours and that deletions are only rarely seen (Supplementary Fig. 14).

## Discussion

Semaphorins constitute a family of secretory or membrane-bound receptors, which were first described as regulators of neuronal axon growth^26^. They are characterized by an extracytoplasmic amino-terminal β-propeller—the SEMA domain—which is needed for plexin receptor binding^27^,^28^. In addition to their role in developmental and physiological processes, semaphorins and their receptors have increasingly been associated with neoplastic disorders (reviewed in refs 24, 26). Interestingly, they have been found to act both, in an anti- and protumoral fashion depending on the particular semaphorin as well as the tumour context. Several tumorigenic properties are thereby influenced including cell proliferation, evasion of apoptosis, angiogenesis, oxidative stress regulation and metastasis. However, no particular semaphorin has been implicated in cancer susceptibility yet. Here we have shown for the first time that *SEMA4A* germline variants predispose to a hereditary neoplastic syndrome.

The germline *SEMA4A* V78M variant was inherited in an autosomal dominant fashion with incomplete penetrance in this family with FCCTX pinpointing additional genetic, environmental or life style modifiers necessary to establish the malignant phenotype. Individuals with this variant developed tumours at a higher age than classical LS patients^7^, showed a moderate number of colorectal adenomas and had a propensity for extracolonic malignancies. Such a genetic modifier might be *MUTYH* where the heterozygous germline variant R217H was found in two *SEMA4A* V78M carriers with CRC (K13, K18). Biallelic germline *MUTYH* mutations—primarily Y179C and G396D—are the cause of *MUTYH*-associated polyposis, which is a rare autosomal recessive syndrome resembling familial adenomatous polyposis^12^,^29^. Monoallelic *MUTYH* germline variants are associated with a small increase in CRC risk; however, this assumption should be handled with care as different studies have come to inconsistent results^12^,^30^,^31^. The *MUTYH* variant R217H found in K13 and K18 has been previously described once in a cohort of 406 patients with more than five polyps and/or CRC from France but its predisposing role has not been established yet^32^.

We were able to identify two further *SEMA4A* variants in a mutational screening of 53 FCCTX patients and studied the segregation of G484A, which followed a dominant inheritance pattern. In both pedigrees, variants were associated with extracolonic neoplasms—ovarian cancer in G484A and endometrial cancer in S326F. Homozygosity of the S326F genotype observed in the index patient could either be the result of an additional germline mutation unrelated to other familial cancer cases or may indicate an autosomal recessive mode of inheritance operational in this family. However, due to lack of DNAs from other family members, we were unable to resolve this issue.

The *SEMA4A* P682S SNP is associated with an increased risk of CRC in our association study including Austrian and German individuals. Although this finding has to be replicated in an independent cohort and might reveal ethnic differences, the data, nevertheless, suggest that P682S constitutes a risk allele for a small proportion of CRC cases probably missed by genome-wide association studies that detect mostly frequent, low penetrant susceptibility loci^2^.

The compound heterozygous germline *SEMA4A* variants D345H and F350C have been described in patients with retinitis pigmentosa and cone rode dystrophy but until now this finding has not been replicated^17^,^33^. Sema4A-deficient mice exhibit photoreceptor degeneration and disturbed T-helper cell function but lack apparently increased tumour development^14^,^34^. Given the wide expression of *SEMA4A* in different tissues, it is plausible that mutations can have different effects depending on the respective tissue. In fact, only the F350C but not the D345H variant was able to recapitulate the retinal disease phenotype of Sema4A-deficient mice in a homozygous knock-in mouse model, a genotype not described in humans yet^18^. This observation stresses the special role of the F350 residue for photoreceptor function. The fact that these mice do not develop overt tumours does not necessarily argue against a potential tumour predisposing role. First, these animals have not been thoroughly investigated for tumour formation, and second, mutations in human cancer susceptibility gene homologues do not consistently result in increased carcinogenesis in mice. With respect to colorectal carcinogenesis, this has been clearly shown for the MMR gene *Pms2* as well as for *Smad4* predisposing to LS and juvenile polyposis syndrome, respectively^35^,^36^. For both conditions, additional germline truncating mutations in the gatekeeper gene *Apc* are needed for intestinal tumour development in mice.

*SEMA4A* variants found in this study were not restricted to a certain hot spot region indicating a loss-of-function mechanism^37^. This assumption is further supported by functional *in vitro* assays performed in the SEMA4A-deficient CRC cell line HCT-116. Whereas activation of mitogenic pathways like MAPK/Erk and PI3K/Akt within these cells could be diminished by transfection of a SEMA4A^WT^ construct, expression of the SEMA4A^V78M^ mutant failed to do so. Accordingly, re-expression of SEMA4A^WT^ but not SEMA4A^V78M^ inhibited G2/M-phase transition in HCT-116, again suggesting a loss-of-function of the V78M substitution. It has to be mentioned that the results of our copy-number analysis demonstrated a gain of chromosome 1q22; however, homozygosity of the V78M variant observed in two of the CRCs could nevertheless indicate that SEMA4A acts as a tumour suppressor rather than a proto-oncogene in the context of familial colorectal tumorigenesis. Middeldorp *et al.*^38^ found that tumour specimens from patients with FCCTX frequently exhibit gains of different chromosomal regions including chromosome 1, which is accompanied by copy-neutral LOH. Loss of the *SEMA4A* wild-type allele accompanied by amplification of the mutant one might be one mechanism of tumour suppressor inactivation in this particular entity. Unfortunately, due to low-quality DNA obtained from formalin-fixed, paraffin-embedded (FFPE) tumour specimens as well as lack of appropriate heterozygous microsatellite loci within or adjacent to the *SEMA4A* gene, we were unable to prove the type of LOH in tumours of Family K. Whether public data indicating that the *SEMA4A* gene is predominantly amplified in diverse cancers can also be interpreted this way, should be handled with extreme caution as context specific functions have to be taken into account. Indeed, it has recently been shown that solubilized Sema4A at high levels is able to suppress cell death induced by plexin D1 in the mouse mammary tumour cell line 4T1, whereas the identical constellation inhibited proliferation in human endothelial cells^16^,^39^.

Our *in vitro* results have shown that SEMA4A^V78M^ differentially modulates the PI3K/Akt and MAPK/Erk pathways in HCT-116 cells and that additional molecular hits are likely needed to establish the SEMA4A^V78M^ phenotype, which is in accordance with well-established concepts of predisposing germline mutations^37^. For instance, mutations in the *PIK3CA* and *KRAS* genes found in the HCT-116 cell line could represent additional oncogenic hits. Recently, two different molecular entities have been postulated among FCCTX families with respect to somatically acquired aberrations found in their CRCs. One entity exhibiting loss of tumour suppressor loci involving the *TP53*, *APC*, *SMAD4* and *DCC* genes as well as mutations in *APC* and *KRAS* and another one with stable genotypes at these loci^40^,^41^,^42^. Although our data demonstrating somatic mutations in the *TP53*, *APC* and *KRAS* genes in CRCs from Family K are in line with these results, the numbers of tumours studied are too small to draw a final conclusion especially with respect to cooperation with SEMA4A^V78M^.

In summary, the data presented here broaden our understanding of the pathophysiological role of semaphorins in human carcinogenesis and will have important consequences for screening and early tumour detection strategies of patients with FCCTX and their family members.

## Methods

### Subjects and primary samples

The study was approved by the institutional review board of the Medical University of Graz, Graz, Austria (MUG) and conducted according to the declaration of Helsinki. Written informed consent was obtained from each study participant or, in the case of deceased patients, close relatives for providing personal and family history data as well as biological specimens. Some of them were processed and stored by the Biobank of MUG.

Family K (germline *SEMA4A* p.Val78Met) was from southern Austria and consisted of 88 members spread into two branches. Clinical data revealed that AC-I criteria compatible with HNPCC (LS) were fulfilled (Fig. 1a; Supplementary Fig. 1). However, CRCs from two patients in either branch (K13 and K26) showed normal expression of the DNA MMR genes *MLH1*, *MSH2*, *MLH6* and *PMS2* by immunohistochemistry as well as microsatellite stability. Furthermore, in individual K13, tumour tissue revealed absence of a somatically acquired *BRAF* mutation. Large germline rearrangements in the MMR genes and *EPCAM*, respectively, as a rare cause of HNPCC were also excluded by multiplex ligation-dependent probe amplification in this patient. We, therefore, made a diagnosis of FCCTX in this family.

To prove that *SEMA4A* germline mutations are also operable in other patients with FCCTX, we studied a cohort of 53 further cases with this syndrome (Supplementary Table 3). Clinical data as well as DNA extracted from PB were provided by the German HNPCC Consortium, Bonn, Germany (*n*=44), the Division of Gastroenterology, Baylor University Medical Center, Dallas, Texas, USA (*n*=6) and the Institute of Human Genetics, MUG (*n*=3) in accordance with local ethical guidelines. One patient (MUG1) fulfilled the modified AC criteria^4^, whereas all others, classical AC-I and/or AC-II criteria.

Index patient BN01 with germline *SEMA4A* p.Ser326Phe mutation had microsatellite stable sigmoid colon cancer. Index patient BN04 identified to carry the germline *SEMA4A* p.Gly484Ala mutation has been analysed for germline mutations in *MLH1*, *MSH2*, *MLH6* and *PMS2* by direct sequencing which revealed negative results. Her brother's CRC (III:IV) showed low microsatellite instability.

### DNA and RNA isolation

DNA purification from PB mononuclear cells, cell lines and fresh frozen tissue specimens were accomplished with the QIAamp DNA Mini Kit (Qiagen) according to the manufacturer's instructions. The RNeasy Mini Kit (Qiagen) was used for RNA isolation from PB mononuclear cells and cell lines. To investigate tumour-specific aberrations, tumour-bearing tissue was manually microdissected from archival, FFPE specimens and DNA isolated using the ReliaPrep FFPE gDNA Miniprep kit (Promega).

### Genotyping and LA

LA was performed in Family K afflicted with FCCTX (Fig. 1a; Supplementary Fig. 1). The GeneChip Human Mapping 250 K Nsp Array (Affymetrix) was used for genotyping of family members according to the manufacturer's protocols. A genome-wide analysis of linkage was conducted under the assumption of an autosomal dominant mode of inheritance with assignment of phenotype to persons affected by the trait (K3, K6, K10, K14, K13, K18) and with additional inclusion of one spouse of one affected person (K15) for improved haplotype reconstruction. The disease allele was assigned a frequency of 0.001 and 100% penetrance for multipoint parametric LA on this subset of family members which was performed with the MERLIN programme in the Alohomora Linkage software tool^43^,^44^.

### Whole exome sequencing and data analysis

WES and analysis were performed in four members of the family (K3, K13, K14, K18). Each patient DNA was prepared according to the Illumina protocols. Briefly, 1 μg of genomic DNA was fragmented and Illumina adaptors were ligated to the fragments. Selected DNA fragments with a size of 350 to 400 bp were then PCR amplified using the TruSeq DNA Sample Preparation kit (Illumina), and the final products were analysed for integrity by the Agilent Bioanalyzer. Multiple DNA libraries were combined with different indices into a single pool before enrichment. Hybridization with capture probes, washing and eluting were performed two times. Enriched targeted regions were amplified by PCR using the same primers from the TruSeq DNA Sample Preparation kit and then sequenced on a HiSeq 2000 Sequencer (Illumina).

Sequence data in FastQ format were aligned to the hg19 version of the human genome (GRCh37) using the Burrows-Wheeler Aligner^45^ (BWA; http://bio-bwa.sourceforge.net/), transformed into SAM files and then converted into compressed BAM files by picard ( http://picard.sourceforge.net/). Possible PCR duplicates were marked by picard and local realignment around indels was performed using the Genome Analysis Tool Kit^46^ (GATK; http://www.broadinstitute.org/gatk/download) to prevent false positive SNPs at the end of sequence reads. GATK was also used to reevaluate base quality scores, perform the raw SNP calling of all sequences within RefSeq gene exons ( http://www.ncbi.nlm.nih.gov/RefSeq/) - plus ten bp at each splice site—and to recalibrate variant quality scores.

With a read length of 101 bp, there were, on average, 88,333,643 total reads that could be mapped to the human genome in 64.5%, respectively. The mean read depth of target regions (96.4% of RefSeq (refGene) coding exons and 97.2% of CCDS coding exons, respectively) was 49.1 ×. The mean coverage of target regions more than 1 × was 94.5% and the mean coverage of target regions more than 10 × was 87.3%, respectively.

Variant calls were annotated with ANNOVAR^47^ ( http://www.openbioinformatics.org/annovar/), which contained the data from dbSNP132 ( http://www.ncbi.nlm.nih.gov/SNP/) and the allele frequencies of the 1000 Genomes Project from February 2012 ( http://www.1000genomes.org/) and of the ESP5400 version of the NHLBI GO Exome Sequencing Project ( https://esp.gs.washington.edu/drupal/). During progression of the study, variants were also manually checked for frequencies in updated versions of those databases. Furthermore, single variants were analysed by the following prediction programs: SIFT ( http://sift.jcvi.org/), Polyphen-2 ( http://genetics.bwh.harvard.edu/pph2/), MutationTaster 2.0 ( http://www.mutationtaster.org/), PhyloP, phastCons and GERP (the last three were precalculated from the UCSC genome browser http://genome.ucsc.edu/cgi-bin/hgGateway). An in-house databank consisting of 18 exomes sequenced on the Illumina platform was used to exclude sequence artifacts as well as variants not covered extensively by public databases. The median age of individuals was 23 years (range 4 to 75 years) and they all are obtained from families lacking a personal or family history of cancer. Variants were excluded if they were found in at least two individuals from the in-house databank, variants found in only one individual were further checked by functional prediction tools.

### Variant resequencing and screening of SEMA4A

Confirmation of mutations detected at WES and screening of the *SEMA4A* gene in 53 further patients with FCCTX were accomplished by PCR and Sanger sequencing. Oligonucleotide primers were designed with Primer-BLAST ( http://www.ncbi.nlm.nih.gov/tools/primer-blast/) or ExonPrimer ( http://ihg.helmholtz-muenchen.de/ihg/ExonPrimer.html), respectively. Primers for resequencing were designed to cover the variant and have a size preferably smaller than 300 bp. All 14 coding exons as well as intron–exon boundaries of the *SEMA4A* gene were analysed. Primers used in this screening are summarized in Supplementary Table 6. They were tagged by M13 sequences to facilitate direct sequencing. PCRs were performed using the HotStarTaq DNA Polymerase (Qiagen) or the peqGOLD Hot Start Mix S (PEQLAB), respectively. Capillary electrophoresis was performed on ABI PRISM 3730 DNA Analyzer or ABI PRISM 310 Genetic Analyzer, respectively (both by Applied Biosystems). Chromatograms were analysed with FinchTV v.1.4.0 (Geospiza) and SeqScape software v.2.5 (Applied Biosystems).

### Reverse transcription and SEMA4A cDNA amplification

RNA (1 μg) was digested with DNase I, RNase-free (Thermo Scientific) and reversely transcribed with random hexamer primers using the RevertAid H Minus First Strand cDNA Synthesis Kit (Thermo Scientific). A negative control (RT-minus) was always included. Primers for amplification of the reference gene *B2M* were as previously described^48^. Primers for *SEMA4A* transcript variants were as follows: var1-3fw, 5′-CCTGGGCCTTTTCCTCTTCC-3′; var124fw, 5′-TTTCTCCTGAATGGCACCCC-3′; var1-4rv, 5′-TTTTTCTGTCACTGGCTGGC-3′ (the reverse primer was the same for all transcript variants). Primers var1-3fw and var1-4rv were also used for direct sequencing of amplified cDNA to assess mRNA expression of the V78M variant.

### Genotyping of *SEMA4A* Pro682Ser

We determined the frequency of *SEMA4A* P682S in a normal Caucasian population and performed a genetic association analysis. Genotypes were determined by a 5′-exonuclease assay (TaqMan). Primer and probe sets were designed and manufactured using Applied Biosystems 'Assay-by-Design' custom service (Life Technologies, USA). General TaqMan reaction conditions were set according to the manufacturer's instructions. Endpoint fluorescence was measured by the POLARstar plate reader (BMG Labtech). The data were exported into an Excel format and depicted and analysed as scatter plot. In this plot, genotype groups were identified as separate and distinguishable clusters. As a control for consistency of genotyping methods, determination of genotypes was repeated in at least 10% of the samples and no discrepancies were observed. Fisher's exact test was used to test for association of genotypes from cases with genotypes from controls (GraphPad Quickcalc online; http://graphpad.com/quickcalcs/contingency1.cfm). Hardy–Weinberg equilibrium testing of cases and control was performed as previously described^49^. Odds ratios were calculated using MedCalc ( http://www.medcalc.org/calc/odds_ratio.php).

### Somatic cancer gene mutation screening

Selected target regions of 50 tumour-associated genes, corresponding to 2,855 COSMIC annotated hot spot mutations, were amplified by multiplexed PCR using the IonAmpliSeq Cancer Hotspot Panel v2 (Thermo Fisher Scientific). Library preparations were performed using the Ion AmpliSeq Library Kit 2.0 (Thermo Fisher Scientific). Emulsion PCR and sequencing were performed with the appropriate kits (Ion One Touch Template Kit v2 and Ion Proton 200 Sequencing Kit, (both from Thermo Fisher Scientific), respectively) on an Ion Torrent Proton sequencer using a single P1 semiconductor chip yielding reads ranging from 90 to 130 bp consistent with the expected PCR fragment size-range. On average, one million reads were obtained for each sample with more than 90% of bases above AQ20 and 87 to 93% reads on-target. Sequence information was obtained from tumour samples in duplicates and additionally from normal non-tumour material.

Initial data analysis was performed using the Ion Torrent Suite Software (Thermo Fisher Scientific, open source, GPL, https://github.com/iontorrent/). Briefly, this included base calling, alignment to the reference genome (hg19) using the TMAP mapper and variant calling with a modified diBayes approach taking into account the flow space information. All called variants were annotated using open source software^47^,^50^ (ANNOVAR, http://www.openbioinformatics.org/annovar/; SnpEff, http://snpeff.sourceforge.net/) and custom Perl scripts. Coding, non-synonymous sequence variations that were detected and confirmed in tumours but not in the normal tissue were further evaluated and visually inspected in IGV ( http://www.broadinstitute.org/igv/) to exclude erroneous variant calls resulting from PCR artifacts or sequence effects. The detection threshold was set to 10% mutated alleles in both duplicates.

### Array comparative genomic hybridization

Tumour DNA samples were labelled using the BioPrime Array CGH Genomic Labeling System (Invitrogen) according to manufacturer's protocol. Briefly, 250 ng of AluI and RsaI digested tumour and reference DNA (Promega) were differentially labelled with dCTP-Cy5 and dCTP-Cy3, respectively (GE Healthcare) and purified by Amicon Ultra-0.5 30kDA filters (Millipore). Analysis of DNA copy-number changes was conducted using a SurePrint G3 60K array (Agilent) scanned on the microarray scanner G2505B (Agilent) according to the manufacturer's instructions. Feature Extraction and DNA Workbench softwares (Agilent) were used for data analysis.

### Digital PCR

The *SEMA4A* V78M mutation was quantitatively analysed with digital PCR (dPCR) on the QuantStudio 3D platform (Life Technologies). A Custom TaqMan SNP Genotyping Assay specific for the analysis of the V78M mutation was used and tested on a StepOne Plus instrument (Life Technologies) using the TaqMan Genotyping Master Mix (Life Technologies) according to the manufacturer's recommendations. For dPCR, 17.4 μl of Digital PCR Master Mix (2 × ) was mixed with 1.7 μl of the TaqMan assay and 60 ng of DNA to a final volume of 36 μl and subjected to two Digital PCR 20k Chips. The chips were thermally cycled in a two-step PCR using the GeneAmp PCR System 9700 (10 min 96 °C, followed by 44 cycles of 56 °C 2 min and 94 °C 30 s, final extension of 2 min 58 °C) and imaged in the QuantStudio 3D instrument. Raw data were analysed using the Relative Quantification module of the QuantStudio 3D AnalysisSuite Software. The confidence level was set to 95% and the desired precision value was 10%.

### Cell culture

Adherent cell lines HT-29, SW-480, HCT-116, HRT-18 and 293T were obtained from ATCC and cultivated for a maximum of 6 weeks in DMEM (Sigma-Aldrich) supplemented with 10% (v/v) HyClone fetal bovine serum (Thermo Scientific) and 1X Antibiotic-Antimycotic (Life Technologies) in a humidified chamber at 37 °C and 5% CO_2_. The identity of all cell lines was confirmed by VNTR analysis using the AmpF/STR Profiler Plus Kit and ABI PRISM 310 Genetic Analyzer (both by Applied Biosystems, respectively) according to the manufacturer's protocols and verified at the online service of the DSMZ cell bank ( http://www.dsmz.de).

### Vectors and transfection

pReceiver-M46 (C-Flag+IRES-eGFP) control, *SEMA4A* wild-type and *SEMA4A* V78M mutated vectors were purchased from GeneCopoeia and propagated in One Shot TOP10 Chemically Competent *E. coli* (Life Technologies). Plasmids were purified by JETSTAR Maxi Plasmid Purification Kit (Genomed) and checked by direct sequencing. One day before transfection, 6 × 10^5^ cells were seeded into six-well tissue culture plates to achieve 60 to 80% confluency. Plasmid and Lipofectamine LTX (Life Technologies) were diluted at a ratio of 1:10 (HCT-116) or 1:5 (293T), respectively, in 500 μl serum-free Opti-MEM medium (Life Technologies) for transfection. If not indicated otherwise, cells were usually grown for 48 h after transfection before whole-cell lysate preparation.

### Whole-cell lysates and immunoblotting

Protein preparations were performed at 4 °C. After washing cells two times with PBS, whole-cell lysates were produced from culture dish attached adherent cells using RIPA Buffer (Sigma-Aldrich) supplemented with 2 × Halt Protease Inhibitor and 2 × Halt Phosphatase Inhibitor Cocktails (Thermo Scientific) which were added just before lysis. Adherent cells were scraped from the plate after incubating on a shaker for 5 to 15 min and subsequently quickfrozen in liquid nitrogen and submitted to two freeze-thaw cycles. Lysate were clarified by centrifugation at 8,000 *g* for 10 min.

Protein concentration was determined with the DC Protein Assay (Bio-Rad) using SPECTROstar Omega and MARS Data Analysis Software (both BMG LABTECH). Lysates were diluted with 4 × Laemmli sample buffer (Bio-Rad) and 710 mM final β-mercaptoethanol and incubated for 5 min at 95 °C. SDS-Polyacrylamide gel electrophoresis of equal protein amounts was performed with precast Mini-PROTEAN TGX 4-15% gels (Bio-Rad). Proteins were blotted onto low fluorescence PVDF transfer (Advansta) or Supported Nitrocellulose (Bio-Rad) membranes, respectively. Membranes were blocked with 3% (wt/v) Non-Fat Dry Milk in TBS (Bio-Rad) with 0.01% (v/v) Tween 20 (Sigma-Aldrich). Proteins were detected with specific primary antibodies directed at: SEMA4A (1:200, #sc-67073, Santa Cruz Biotechnology), Active-β-Catenin (1:1,000, #05-665, Millipore), β-Catenin (1:200, #sc-1496, Santa Cruz Biotechnology), Akt (pan) (1:1,500, #4691, Cell Signaling), Phospho-Akt (Ser473) (1:2,000, #4060, Cell Signaling), p44/42 MAPK (Erk1/2) (1:2,500, #4695, Cell Signaling), Phospho-p44/42 MAPK (Erk1/2) (Thr202/Tyr204) (1:2,000, #4370, Cell Signaling), GSK-3β (1:1,500, #9832, Cell Signaling), Phospho-GSK-3β (Ser9) (1:3,000, #5558, Cell Signaling), GAPDH (1:2,000, #sc-32233, Santa Cruz Biotechnology). Horseradish peroxidase-linked secondary antibodies were anti-rabbit IgG (#7074, Cell Signaling) and anti-mouse immunoglobulins (#P026002, Dako), both diluted 1:10,000, respectively. Membranes were incubated in Restore Plus Western Blot Stripping Buffer (Thermo Scientific) at 37 °C to strip antibodies. Imaging of blots was performed by chemiluminescence using WesternBright ECL horse radish peroxidase substrate (Advansta), CL-XPosure films (Thermo Scientific) and CURIX 60 developer (Agfa Healtcare), respectively. ImageJ 1.47v (NIH, rsbweb.nih.gov/ij) was used for analysis of band densities.

### Surface expression studies

Analysis of Sema4A surface expression in ARPE-19 cells was performed as previously described^18^. The cDNA sequence encoding full-length mouse Sema4A (amino acids 1–760) was generated by PCR and then ligated into pEGFP-N3 (Clontech, Palo Alto, CA). Mutant Sema4AV78M-EGFP construct was generated from Sema4AWT-EGFP using a QuikChange II XL site-directed mutagenesis kit (Stratagene) according to the manufacturer's protocol. Cells were transfected using FuGENE HD (Roche).

### Migration assay

A cell exclusion zone migration assay was performed with the Radius 24—Well Cell Migration Assay plate (Cell Biolabs) according to the manufacturer's instructions. Briefly, HCT-116 cells were seeded into 60 mm cell culture dishes and transfected with control and SEMA4A vectors. Six hours after transfection, cells from one 60-mm dish were split into four wells of one assay plate and grown overnight to allow attachment at full confluency. Time lapse microscopy was started 24 h post transfection by removing the gel spot and concurrent switching of medium to DMEM with 1% (v/v) fetal bovine serum. Cells were monitored for 48 h with a 1-h interval by the Cell Observer (Carl Zeiss). ImageJ 1.47v was used for analysis of cell migration. Closed areas were calculated for each well at different time points by subtracting the open surface area at a given time point from the open surface area at the beginning of the migration assay.

### 7-AAD/BrdU staining and flow cytometry

Twenty-four hours after transfection in 35 mm dishes as described, 1.5 × 10^6^ HCT-116 cells were transferred to 100 mm cell culture dishes and grown for approximately 24 h under normal conditions. BrdU at a final concentration of 50 μM was then added and cells were incubated for 1 h protected from light to label actively proliferating cells. One million cells were washed with ice cold PBS by centrifugation at 4 °C and then fixed for 30 min at room temperature, permeabilized for 10 min on ice, refixed for 5 min at room temperature, treated with DNase and finally stained with APC anti-BrdU antibody (1:50 for 30 min at room temperature) as well as 7-AAD according to the instructions from the APC BrdU Flow Kit (BD Pharmingen). Unlabelled native cells were used as a negative control for the APC anti-BrdU antibody. Stained cells were acquired on the BD LSR II Flow Cytometer operated with FACSDiva Software (both from BD Biosciences, respectively) with a flow rate of less than 400 cells s^−1^ on the same day of staining. Kaluza Flow Cytometry Analysis Software v1.2 (Beckman Coulter) was used for analysis and illustration of flow cytometry data.

### Multiple sequence alignment and 3D modelling of SEMA4A

Multiple sequence alignment was performed with Clustal Omega ( http://www.clustal.org/omega/). Structural models of SEMA4A containing the SEMA and PSI domains only (amino acids 55 to 527 in NP_001180229.1 reference sequence) were generated using the intensive model algorithm of phyre2 (ref. 51) and drawn by POLYVIEW-3D ( http://polyview.cchmc.org/polyview3d.html).

### Statistics

Results obtained from experiments with isogenic cell lines were compared in Excel 2013 using a paired, two-tailed Student's *t*-test.

## Author contributions

E.S. and H.S. designed the study. E.S., P.K., A.W. and H.S. collected family data. P.K., S.Holzapfel, S.L., C.B.-S., V.S., J.B.G., C.R.B. and H.S. obtained patient samples and clinical data. A.R.J. performed the linkage analysis. P.U. analysed the whole-exome sequencing raw data. E.S., A.L. and S. Hofer performed direct sequencing. E.H. performed and analysed dPCR. K.K. performed targeted deep sequencing and analysis. C.W. provided in-house exome data. W.R. and A.G. performed and supervised genetic association analysis. E.S. and S.N. performed *in vitro* experiments. E.S., W.R., A.Z., A.W., G.H., M.R.S., A.K. and H.S. interpreted results. H.S. oversaw the study. E.S. and H.S. wrote the manuscript which was reviewed and approved by all co-authors.

## Additional information

**Accession codes:** Raw sequencing data have been deposited in the European Genome-Phenome Archive (EGA, http://www.ebi.ac.uk/ega/) under the accession code EGAS00001000957.

**How to cite this article:** Schulz, E. *et al.* Germline variants in the *SEMA4A* gene predispose to familial colorectal cancer type X. *Nat. Commun.* 5:5191 doi: 10.1038/ncomms6191 (2014).

## Supplementary Material

Supplementary Information Supplementary Figures 1-14 and Supplementary Tables 1-6

## Figures and Tables

**Table 1:** Clinical characteristics of colorectal neoplasms of Family K exhibiting the germline V78M *SEMA4A* mutation.

**Patient**	**Neoplasm**	**Age (years)**	**Histology**	**Grading/staging**	**Localization**	***SEMA4A*** **V78M**
K6	CRA	44	Tubular adenoma	Well to moderately differentiated	NA	**+**
K6	CRA	61	Tubular adenoma	Well differentiated	Sigmoid colon	**+**
K10	CRA	66	Tubular adenoma	Well to moderately differentiated	NA	**+**
K13	CRC	48	Adenocarcinoma	pG-3, pT-4, pN-1	Coecum	**+**
K14	CRA	63	Tubular adenoma	Well differentiated	Rectum	**+**
K16	CRC	71	Tubulopapillary and mucinous adenocarcinoma	pG-2, pT-2, N-0	Coecum	**+**
K16	CRA	71	Tubulovillous adenoma	Well to moderately differentiated	Coecum	**+**
K16	CRC	72	Tubular adenocarcinoma	pG-2, pT-X	Descending/sigmoid colon	**+**
K17	CRA	46	Tubular adenoma	Well differentiated	Rectum	−
K18	CRC	62	Tubular adenocarcinoma	pG-2, pT-1, N-0	Sigmoid colon	**+**
K18	CRA	62	Tubular adenoma	Well to moderately differentiated	Sigmoid colon	**+**
K18	CRA	62	Tubular adenoma	Well to moderately differentiated	Sigmoid colon	**+**
K18	CRA	64	Tubular adenoma	Well to moderately differentiated	Ascending colon	**+**
K18	CRA	65	Tubular adenoma	Well to moderately differentiated	Descending colon	**+**
K18	CRA	66	Tubular adenoma	Well to moderately differentiated	NA	**+**
K18	CRA	67	Tubular adenoma	Well to moderately differentiated	Descending colon	**+**
K20	CRA	55	Tubulovillous adenoma	Well differentiated	Sigmoid colon	−
K26	CRC	55	Adenocarcinoma	pG-2, pT-3, pN-2	Rectum	**+**

CRA, colorectal adenoma; CRC, colorectal cancer; NA, not available.

**Table 2:** Results of targeted deep sequencing of cancer hot spot regions in CRCs from Family K with the germline V78M *SEMA4A* mutation.

**Patient**	**Somatic mutation**	**Protein alteration**	**dbSNP141**
K13	*APC* NM_000038.5:c.2626C>T	p.R876X	rs121913333
K13	*APC* NM_000038.5:c.4348C>T	p.R1450X	rs121913332
K13	*KRAS* NM_004985.4:c.34_35delinsAT	p.G12I	NA
K13	*TP53* NM_000546.5:c.380C>A	p.S127Y	NA
K13	*PIK3CA* NM_006218.2:c.1633G>A	p.E545K	rs104886003
K16	None found	None found	—
K18	*TP53* NM_000546.5:c.844C>T	p.R282W	NA
K26	*APC* NM_000038.5:c.4135G>T	p.E1379X	rs121913326
K26	*KRAS* NM_004985.4:c.34G>A	p.G12S	NA
K26	*TP53* NM_000546.5:c.743G>A	p.R248Q	rs11540652

CRC, colorectal cancer; NA, not available.

**Table 3:** Results of the *SEMA4A* Pro682Ser association study.

Cohort*	**No.**	**Genotypes**			**Frequency of allele T (%)**	**Two-tailed** ***P*** **value**†	**OR (95% CI)**
		**CC**	**CT**	**TT**			
German and Austrian FCCTX individuals	47	41	6	0	6.4	0.0008	6.793 (2.634 to 17.518)
Non-cancer controls‡	1,138	1,114	24	0	1.1	—	1

CI, confidence interval; FCCTX, familial colorectal cancer type X; OR, odds ratio.

^*^Genotypes of cases and controls were in Hardy–Weinberg equilibrium, with *P* values (df=1) of 0.718 and 0.647, respectively.

^†^Fisher's exact test of genotype counts from cases versus controls.

^‡^Men, *n*=574 (50.4%); women, *n*=564 (49.6%); mean age: 60 years (±s.d. 18); median age: 64 years (range 15–99).
