# Supplementary material for: Germline variants in the SEMA4A gene predispose to familial colorectal cancer type X
Source: Nat Commun. 2014 Oct 13;5:5191. doi: 10.1038/ncomms6191 (PMC4214414; doi:10.1038/ncomms6191)
Supplement: Supplementary Information — Supplementary Figures 1-14 and Supplementary Tables 1-6 [file ncomms6191-s1.pdf]

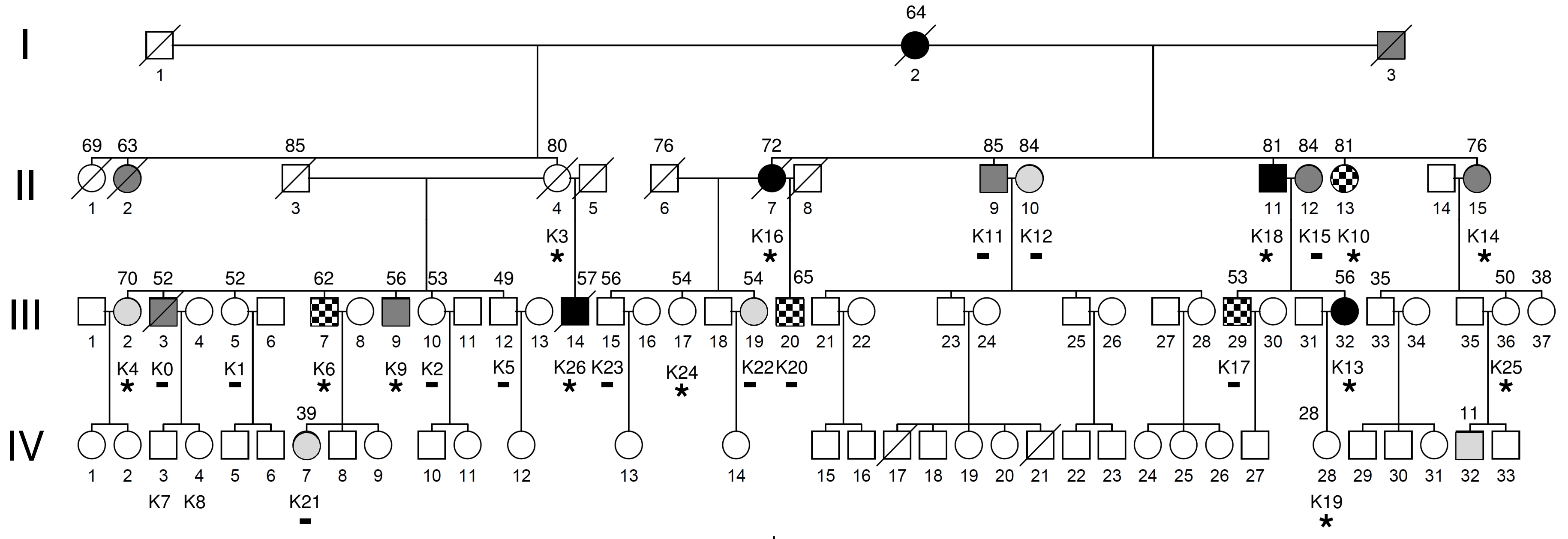

Individual	Neoplasm	Age at diagnosis (in years)
I:2	CRC	n\a
I:3	PC	n\a
II:2	Uterine cancer	31
II:7	CRA, 2 CRCs	71, 71, 72
II:9	Laryngeal cancer	79
II:10	UM	64
II:11	CRC, 6 CRAs, PC, lipoma	62, 62- 67, 74, 75
II:12	RCC	76
II:13	Angiomyolipoma, UM, CRA	53, 57, 66
II:15	Uterine tumor (unclassified), CRA, BC	46, 63, 67
III:2	UM, lipoma	46, 51
III:3	Thyroid adenoma, AML	37, 51
III:7	2 CRAs	44, 61
III:9	Testicular cancer	36
III:14	CRC	55
III:19	UM	43
III:20	CRA	55
III:29	CRA	46
III:32	UM, BC, CRC	32, 47, 48
IV:7	Breast adenoma	23
IV:32	Fetal intracardiac myoma	-

Supplementary Figure 1 | Extended pedigree of Family K. Asterisk, *SEMA4A* V78M carrier; minus, *SEMA4A* wild-type; black symbol, colorectal cancer; checkered symbol, colorectal adenoma; dark gray, malignant neoplasm; light grey, benign neoplasm. BC, breast cancer; CRA, colorectal adenoma; CRC, colorectal cancer; PC, prostate cancer; RCC, renal cell carcinoma; UM, uterine myoma; n\a, not available. Numbers above symbols indicate the age of death or the age at the time last seen (both in years, respectively).

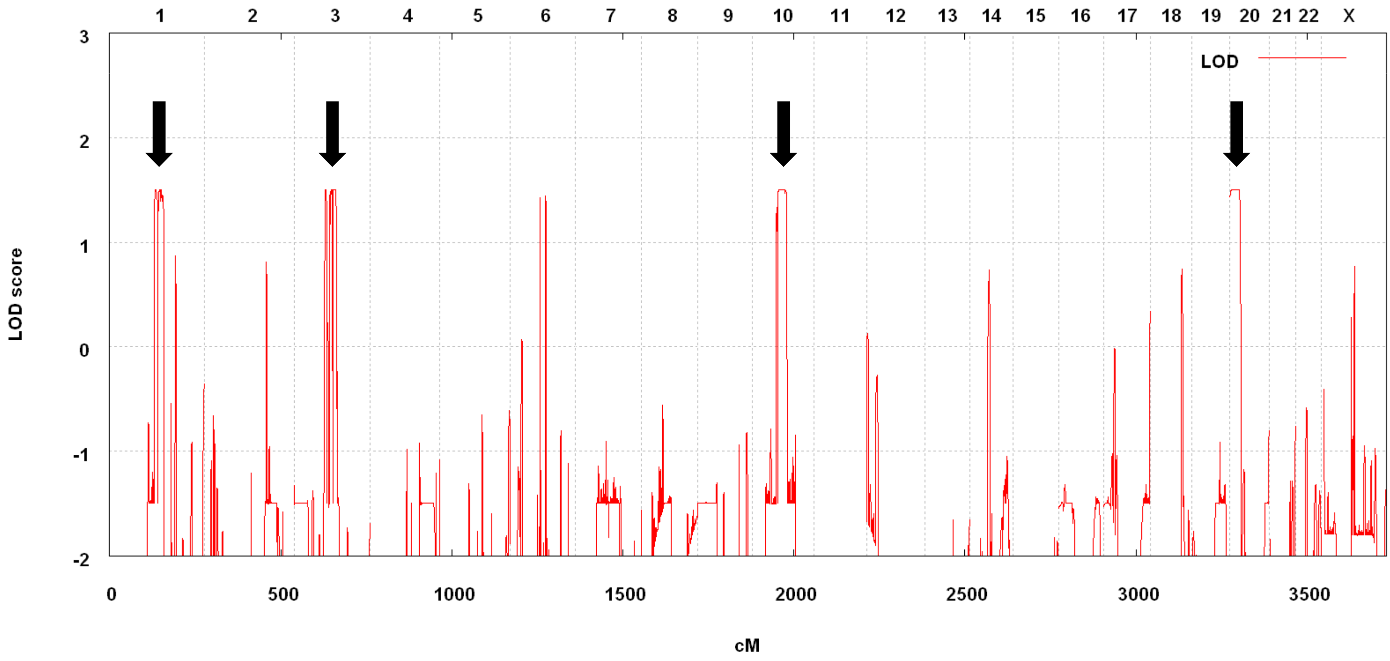

Supplementary Figure 2 | Disease gene mapping in Family K. The panel shows parametric LOD score values in relation to the genetic position identifying four regions (arrows) of the genome reaching the maximal expected LOD score of 1.5. *SEMA4A* is located on chromosome 1q22. Human chromosomes are concatenated from p-ter (left) to q-ter (right) on the x-axis, and the genetic distance is given in centiMorgan (cM).

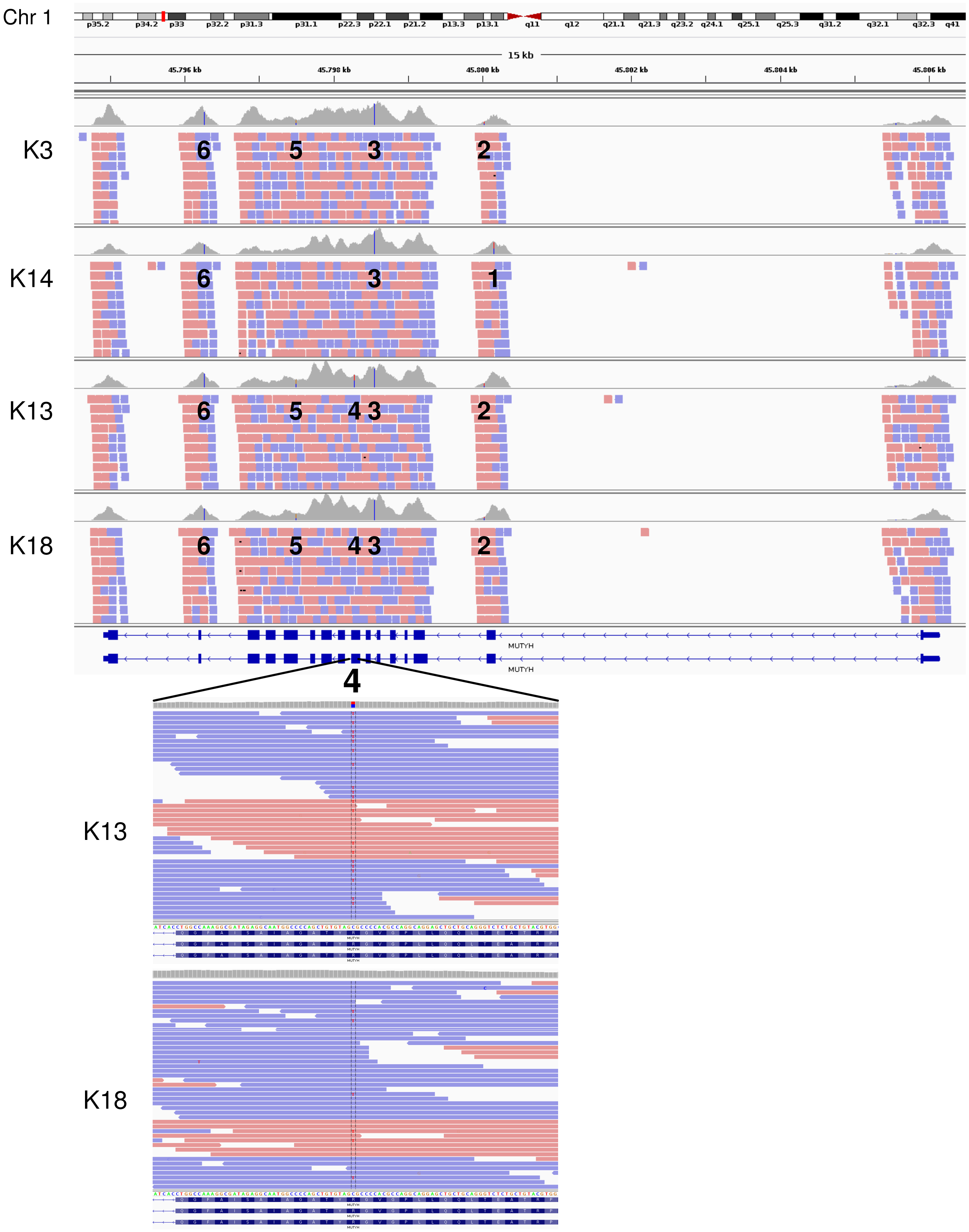

Supplementary Figure 3 | Analysis of the MUTYH gene in four individuals of family K. No additional mutations other than Arg217His were found. The average coverage of the coding regions of *MUTYH* was 58.7x, 93.3x, 56.4x and 95.7x, and the percent coverage ≥ 20x was 92%, 100%, 94% and 100% in the individuals K3, K13, K14 and K18, respectively. The Arg217His variation was seen in 13.25% of reads (coverage 83x) in individual K18 and 45% (60x) in K13, respectively. The sequencing data was visualized using the Integrative Genomics Viewer (IGV; Broad Institute). 1, rs3219484 (Val22Met); 2, rs3219485; 3, rs3219487; 4, rs147754007 (Arg217His); 5, rs3219489 (Glu335His); 6, rs3219493.

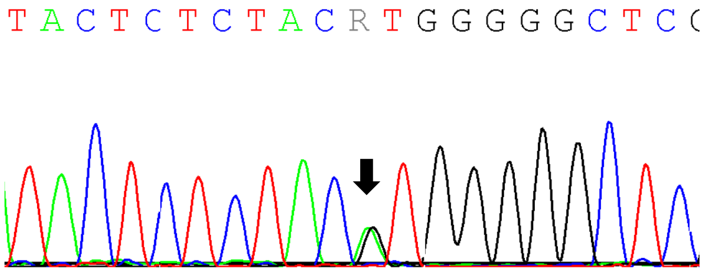

Supplementary Figure 4 | Sanger sequencing of amplified *SEMA4A* cDNA from lymphocytes of individual K13. The V78M mutation (c.232G>A) is normally expressed.

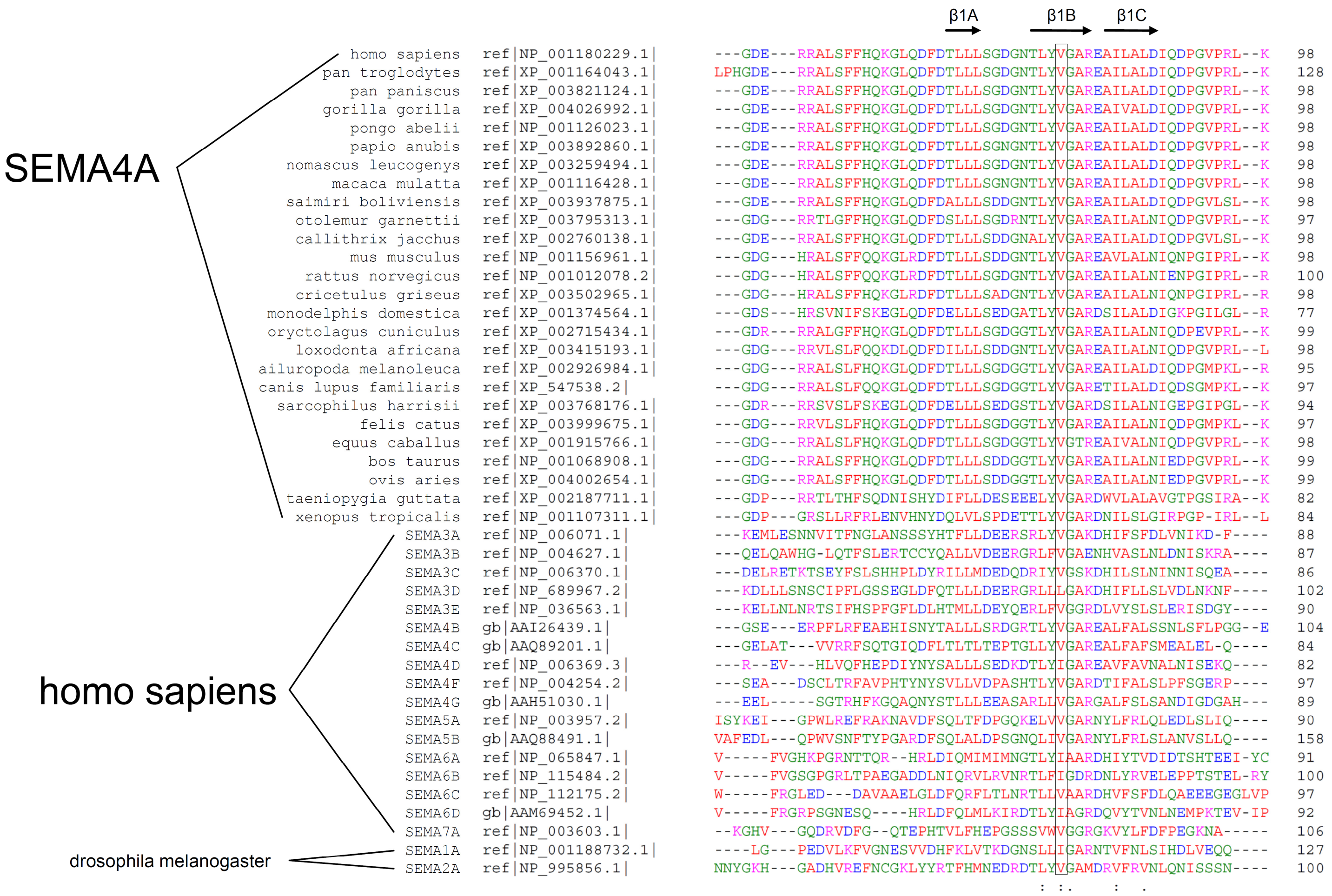

Supplementary Figure 5 | Multiple sequence alignment of semaphorin family members with focus on human SEMA4A Val78. Sequences of SEMA4As found in vertebrates and of all human semaphorins as well as semaphorins found in D. melanogaster were taken from NCBI's RefSeq Protein Database and aligned with Clustal Omega. Human Val78 is identical in all SEMA4As and also conserved in other classes. Secondary structure assignments derived from the crystal structures of Sema4D are adapted from Janssen et. al. (Nature. 2010 Oct 28;467(7319):1118-22. doi: 10.1038/nature09468) and displayed above the alignment where arrows represent β-sheets. The complete depicted sequence segment is part of the SEMA domain. Note that semaphorin classes 3 to 7 can be found in vertebrates only.

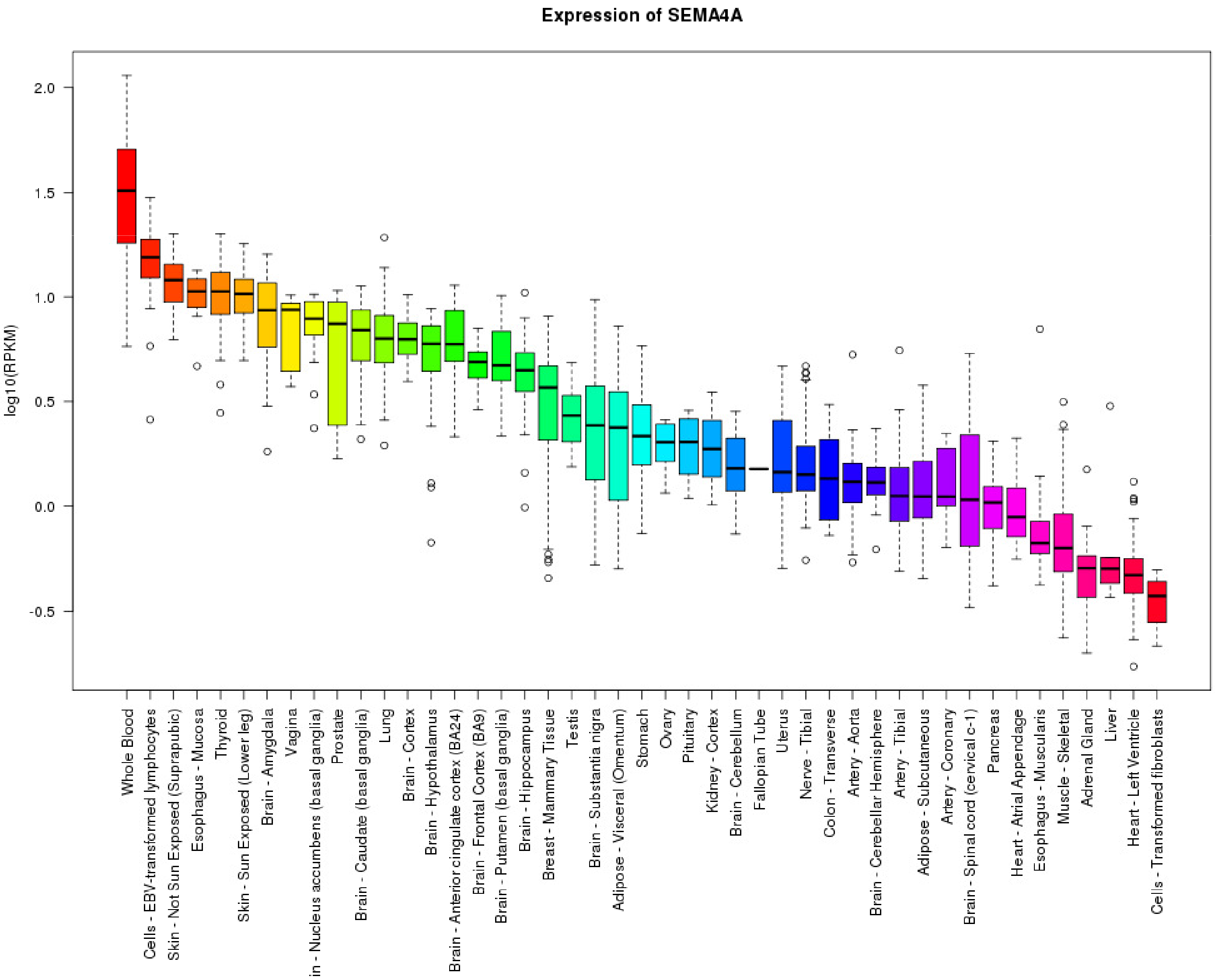

Supplementary Figure 6 | mRNA expression of *SEMA4A* in different human tissues. Data was extracted from the Genotype-Tissue Expression Portal (GTEx; http://www.broadinstitute.org/gtex/).

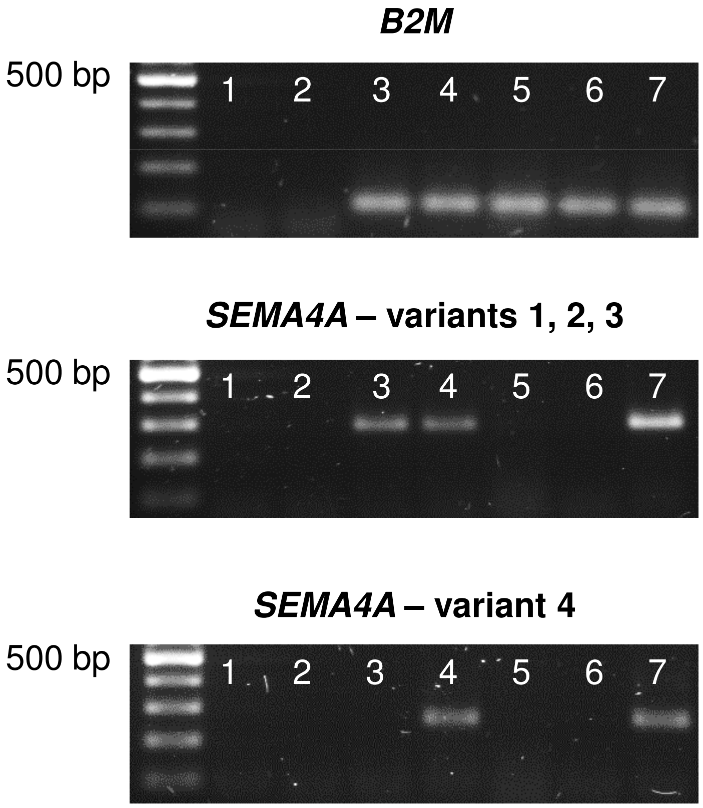

Supplementary Figure 7 | Expression of *SEMA4A* in CRC cell lines . Transcript variants of both *SEMA4A* isoforms were amplified. *B2M* was used as a reference gene. Jurkat cells (acute T-ALL) were used as a positive control. 1, Blank; 2, reverse-transcriptase negative control (Jurkat); 3, Jurkat ; 4, HT-29; 5, SW-480 ; 6, HCT-116; 7, HRT-18 .

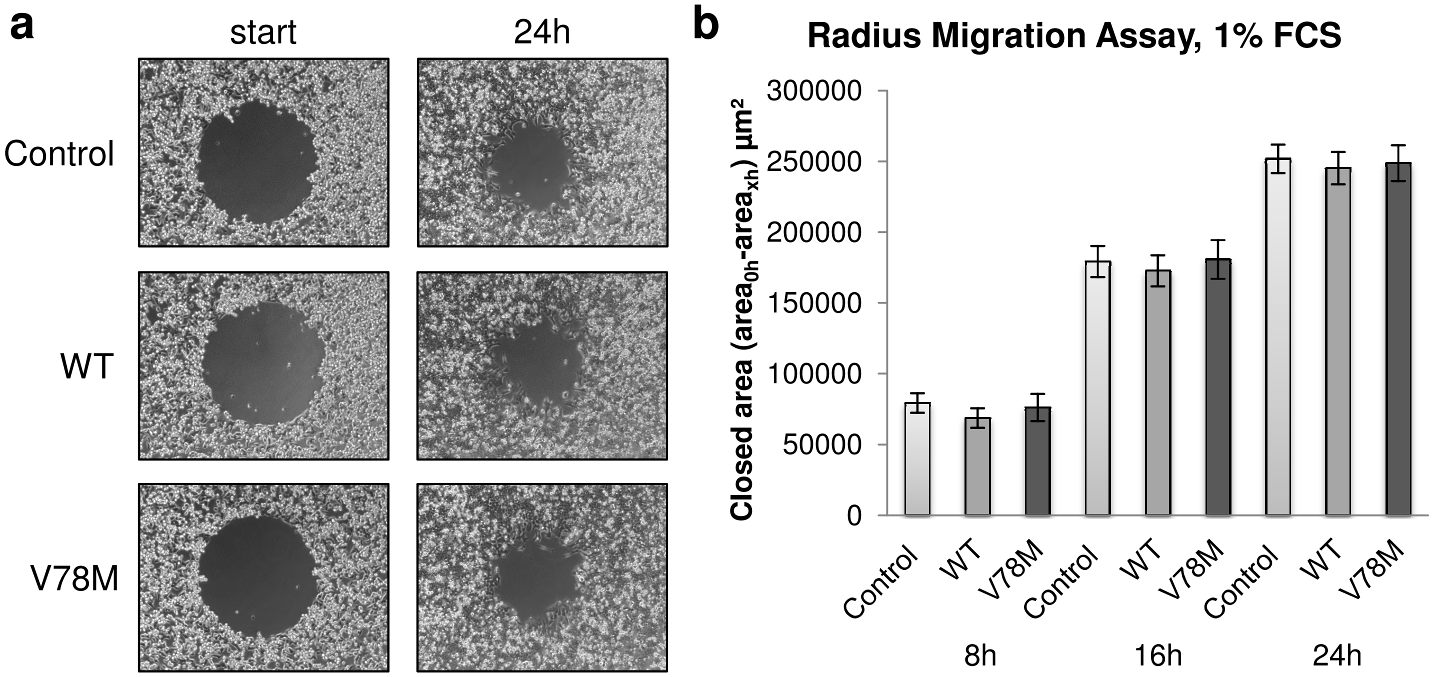

Supplementary Figure 8 | Radius migration assay of *SEMA4A* transfected HCT-116 cells. (**a**) Representative microscopic pictures from one experiment. (**b**) Results of three experiments performed as quadruplicates. Bars represent means ± s.e.m.

Anti-SEMA4A ,1:200

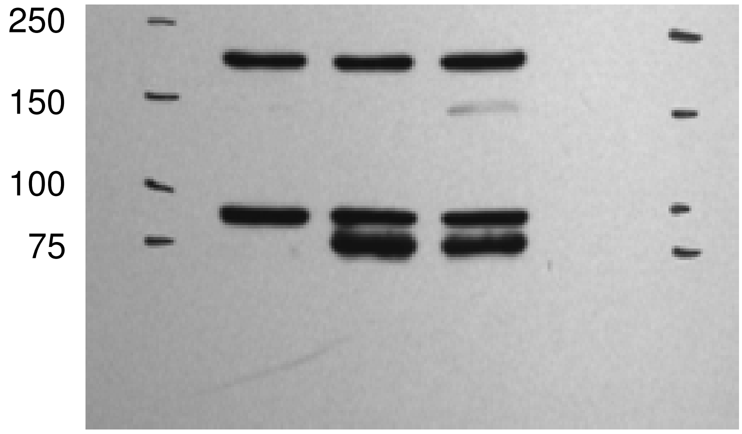

Anti-Akt (pan), 1:1500; Anti-p44/42 MAPK (Erk1/2), 1:2500

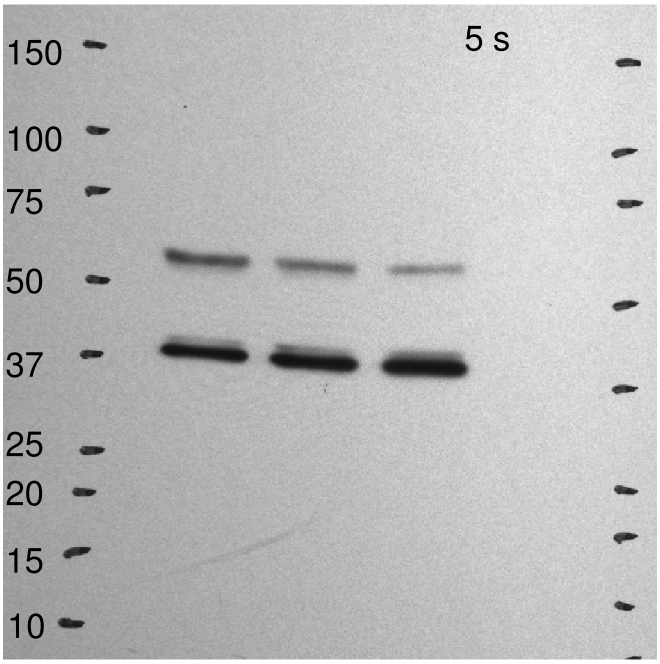

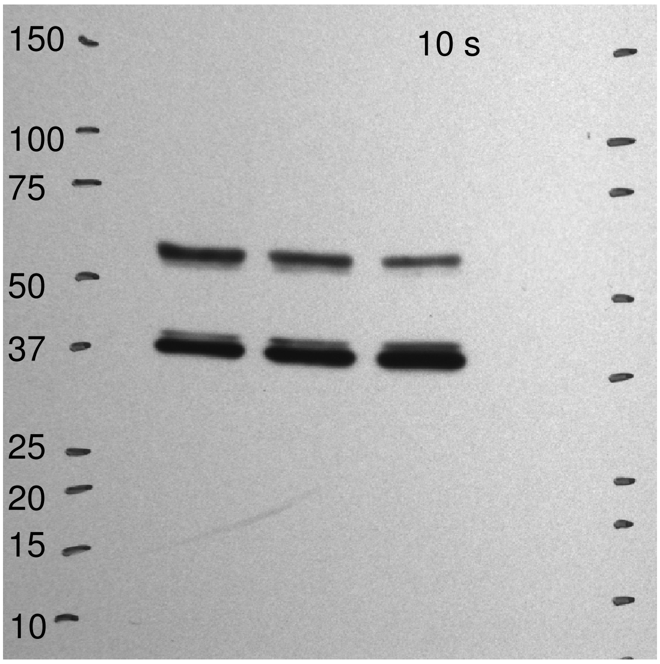

Anti-Phospho-Akt, 1:2000; Anti-Phospho-p44/42 MAPK, 1:2000

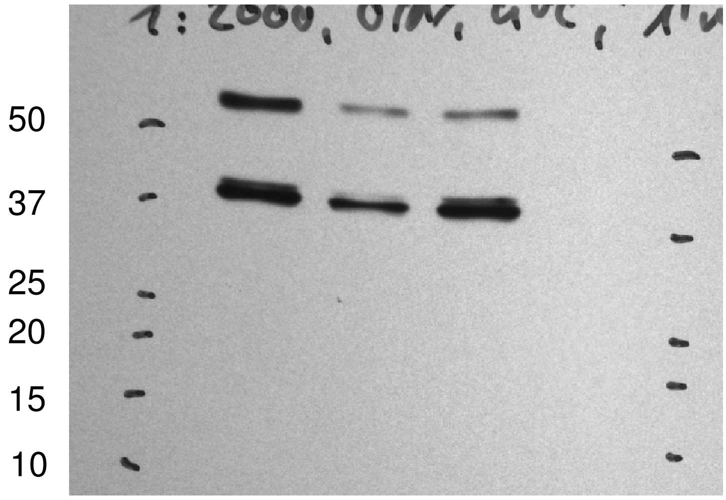

Anti-GAPDH, 1:2000

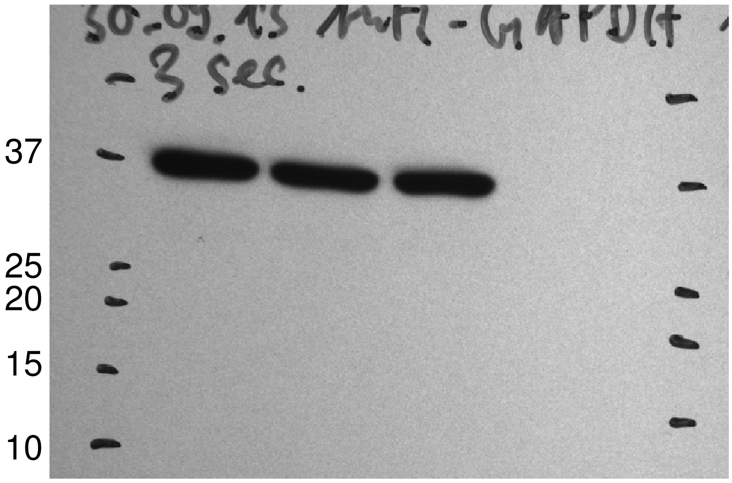

Anti-active-β-Catenin, 1:1000

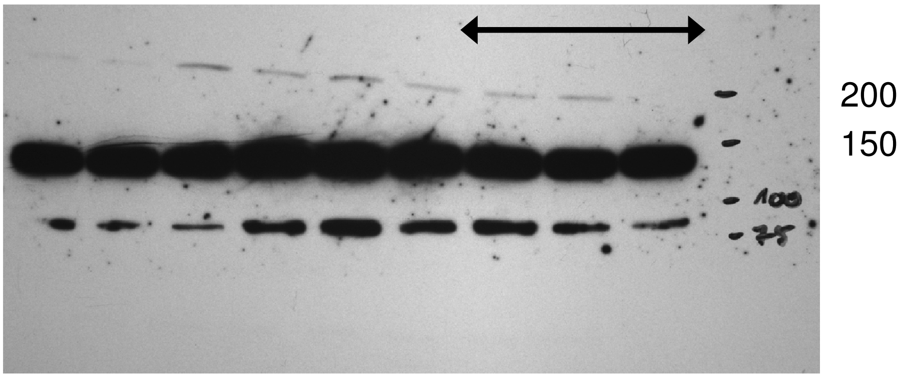

Anti-β-Catenin, 1:200

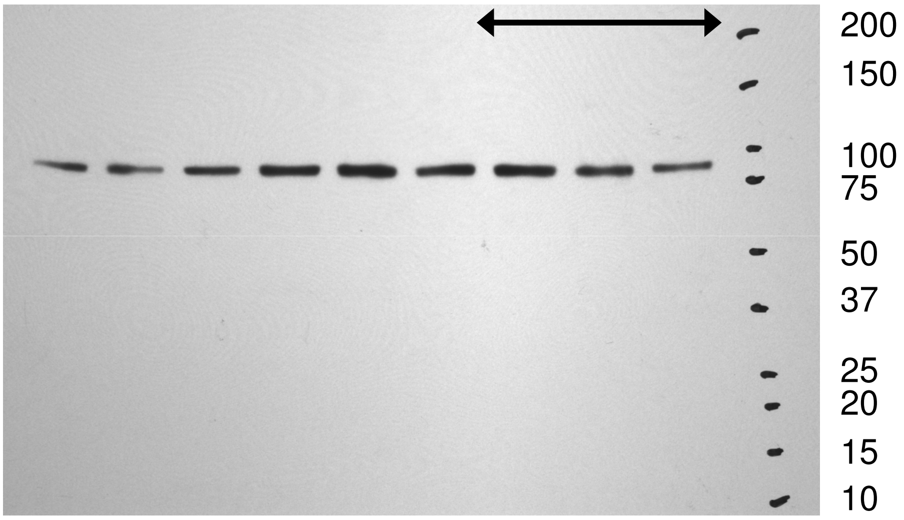

Anti-GSK-3β ,1:1500

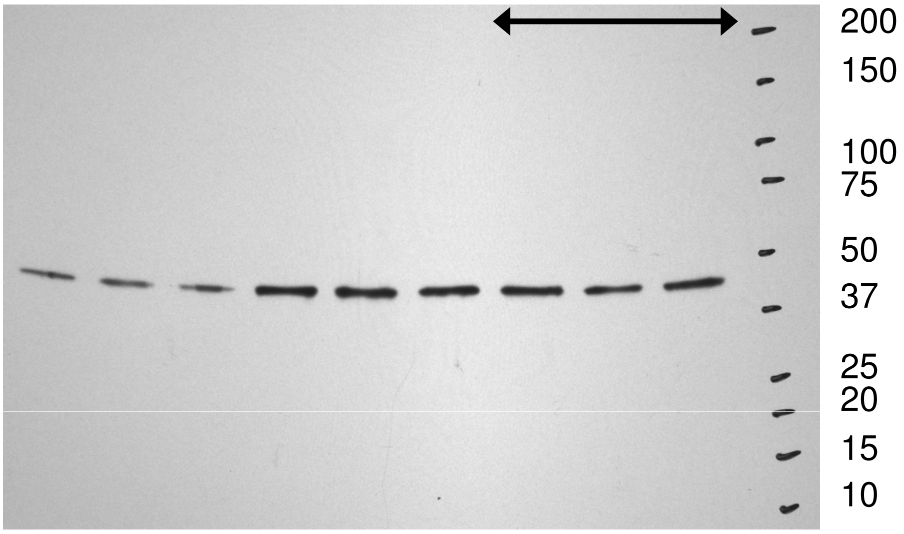

Anti-Phospho-GSK-3β,1:3000

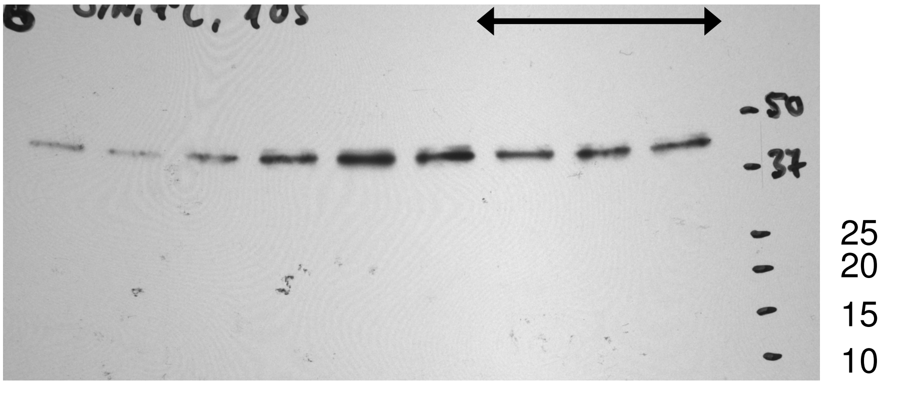

Supplementary Figure 9 | Uncropped blots of Figure 4d.

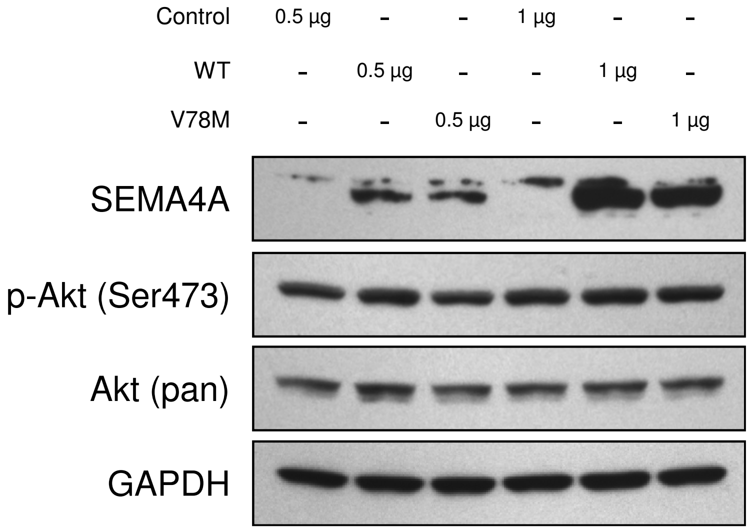

Supplementary Figure 10 | Immunoblots of *SEMA4A* transfected 293T cells showing a representative result of two independent experiments. No effects on PI3K/Akt pathway were seen after transfection of *SEMA4A*. The MAPK/Erk pathway is not constitutively active in this cell line.

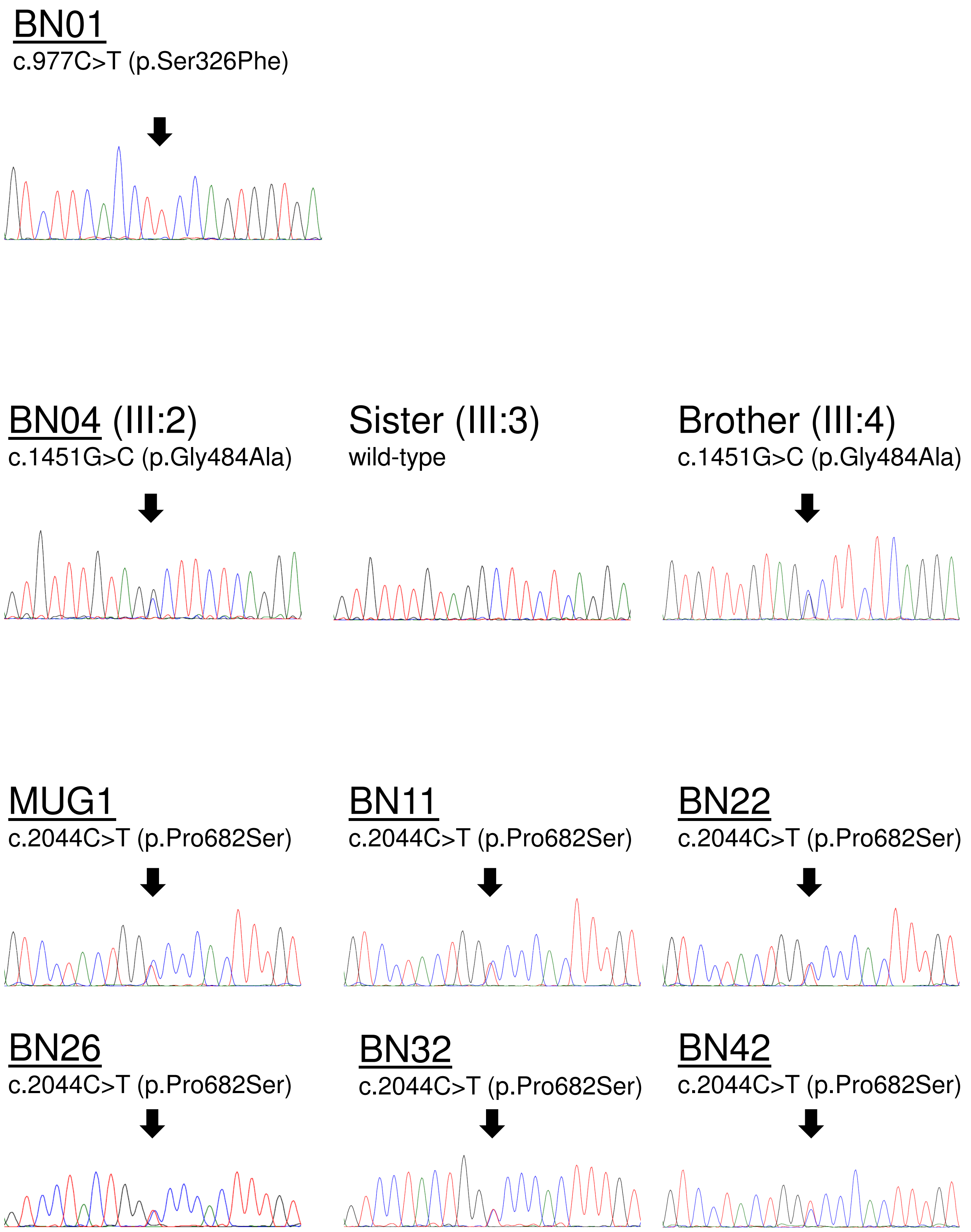

Supplementary Figure 11 | Further *SEMA4A* germline variants identified in a screening of FCCTX patients.

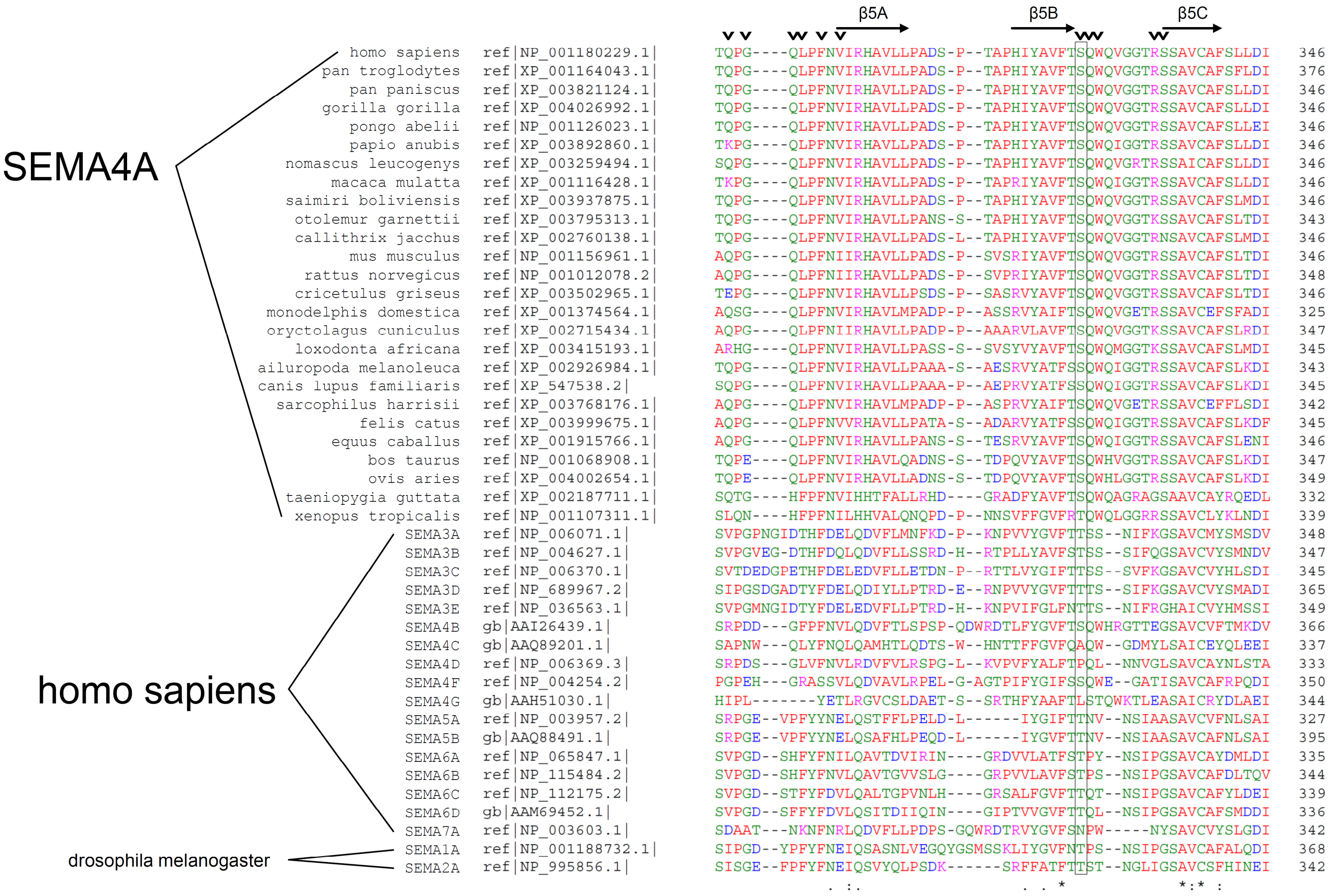

Supplementary Figure 12 | Multiple sequence alignment of semaphorin family members with focus on human SEMA4A Ser326. Sequences of SEMA4As found in vertebrates and of all human semaphorins as well as semaphorins found in D. melanogaster were taken from NCBI's RefSeq Protein Database and aligned with Clustal Omega. Secondary structures assignments derived from the crystal structures of Sema4D are adapted from Janssen et. al. (Nature. 2010 Oct 28;467(7319):1118-22. doi: 10.1038/nature09468) and displayed above the alignment. Arrows represent β-sheets and arrowheads indicate residues involved in homodimer formation (adapted from NCBI's RefSeq Protein Database). The complete depicted sequence segment is part of the SEMA domain. Note that semaphorin classes 3 to 7 can be found in vertebrates only.

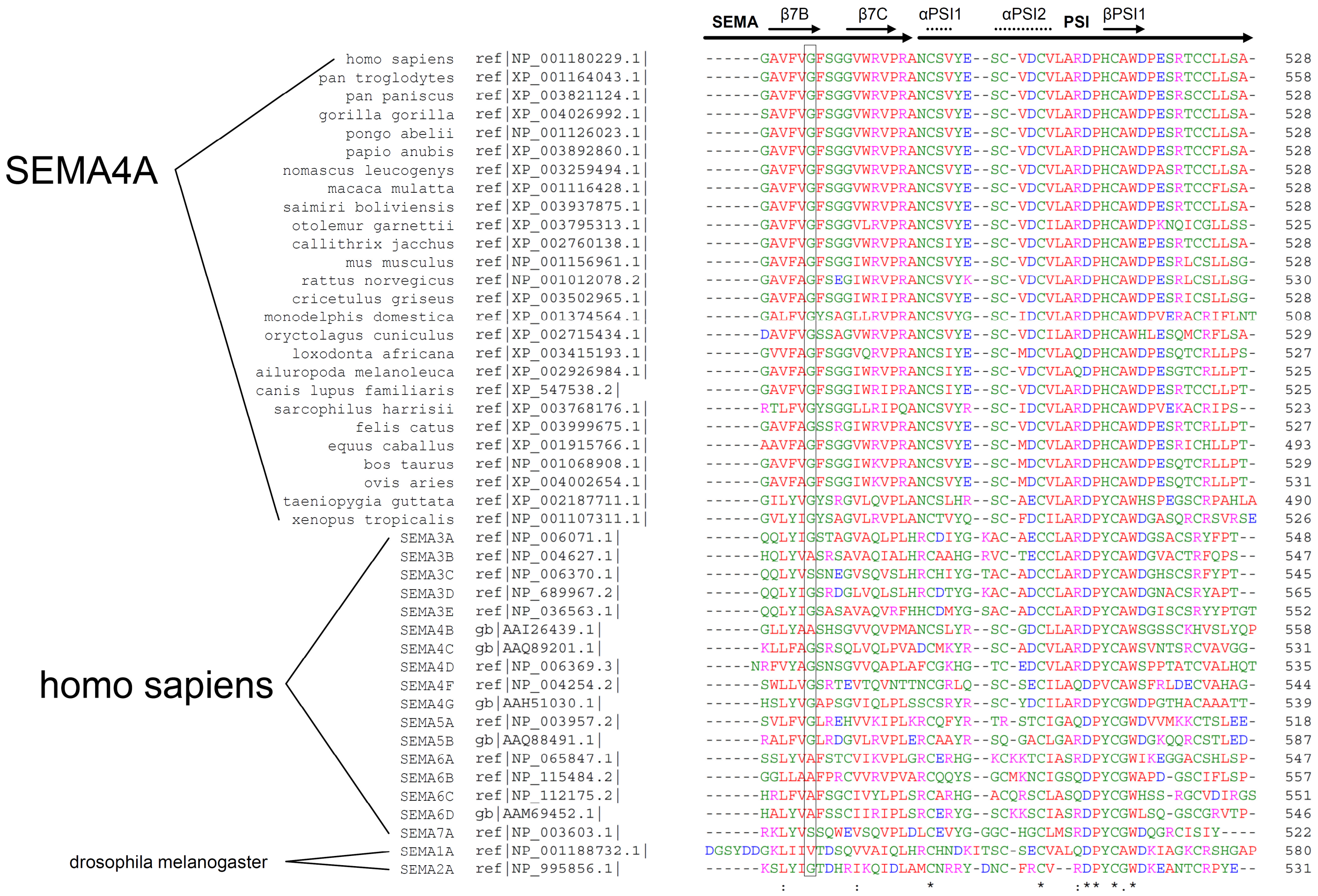

Supplementary Figure 13 | Multiple sequence alignment of semaphorin family members with focus on human SEMA4A Gly484. Sequences of SEMA4As found in vertebrates and of all human semaphorins as well as semaphorins found in D. melanogaster were taken from NCBI's RefSeq Protein Database and aligned with Clustal Omega. Human Gly484 is identical in known SEMA4As. Secondary structures assignments derived from the crystal structures of Sema4D are adapted from Janssen et. al. (Nature. 2010 Oct 28;467(7319):1118-22. doi: 10.1038/nature09468) and displayed above the alignment. Small arrows represent β-sheets and dotted lines α-helices. Note that semaphorin classes 3 to 7 can be found in vertebrates only.

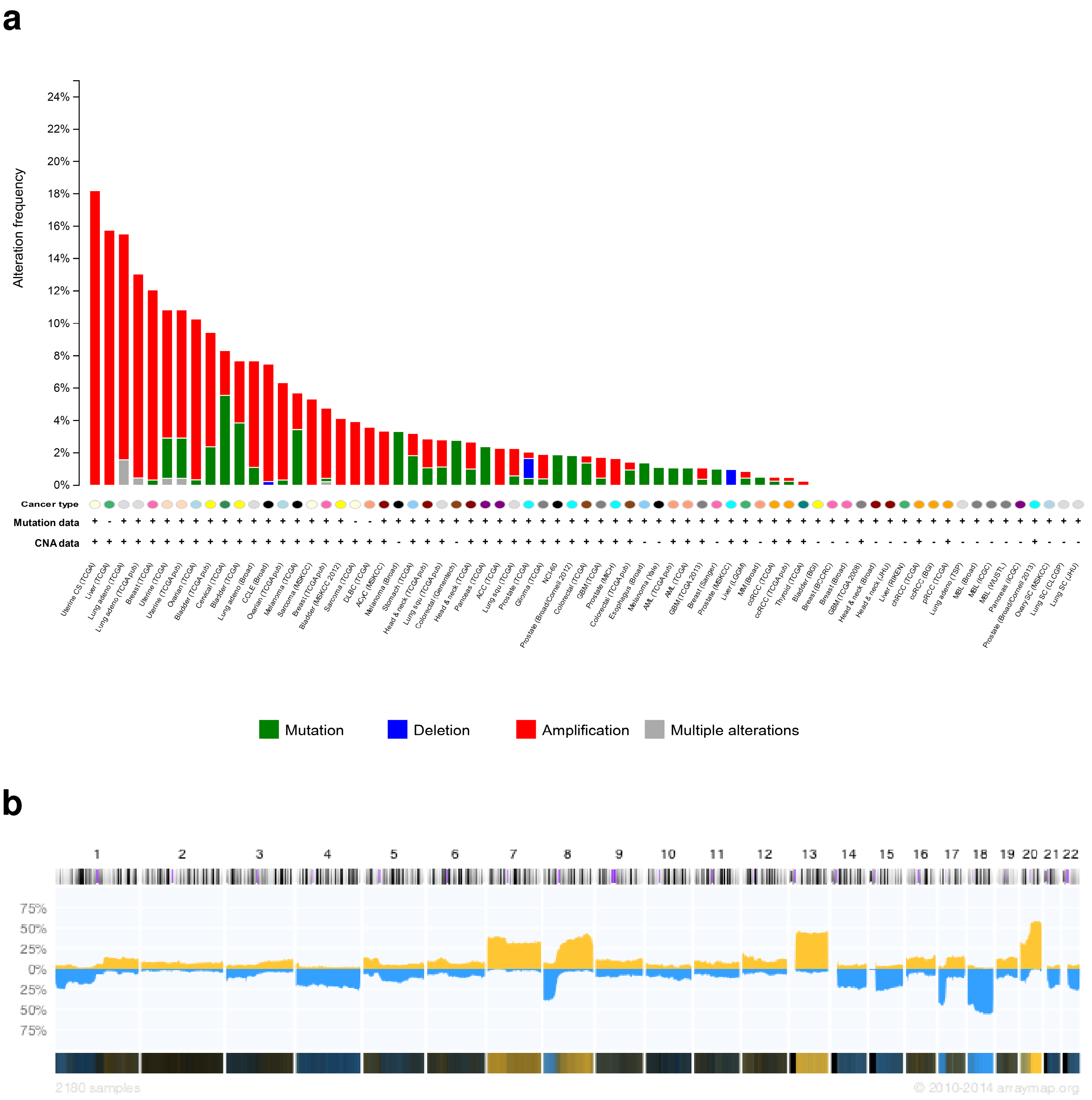

Supplementary Figure 14 | Deletions are rarely seen in *SEMA4A*. (**a**) Cross cancer alterations of *SEMA4A*. Data was extracted from cBioPortal for Cancer Genomics (http://www.cbioportal.org/public-portal/) on 24. March 2014. (**b**) Merged aCGH data of 2180 CRC samples with matched criteria from 34 publications showing gains of chromosome 1q in more than 10% of cases. Data stems from Progenetics (www.progenetics.com) extracted on 26. June 2014.

Supplementary Table 1 | All shared heterozygous variations of individuals K3, K13, K14 and K18 from the linkage

Gene	ExonicFunc	AAChange	dbSNP141	Chr	Start	End	Ref	Obs
ADAMTSL4	nonsynonymous SNV	NM_019032:c.3179G>A:p.R1060H	rs147697821	chr1	150532626	150532626	G	A
CDC25B	nonsynonymous SNV	NM_004358:c.400C>T:p.R134C	rs147172963	chr20	3781128	3781128	C	T
DCBLD2	nonsynonymous SNV	NM_080927:c.1247T>C:p.V416A	n\a	chr3	98531292	98531292	A	G
GRAMD1C	nonsynonymous SNV	NM_001172105:c.472A>C:p.I158L	rs78547874	chr3	113634682	113634682	A	C
LNP1	nonsynonymous SNV	NM_001085451:c.190T>C:p.C64R	rs75122231	chr3	100170596	100170596	T	C
LNP1	nonsynonymous SNV	NM_001085451:c.194A>T:p.H65L	rs76354691	chr3	100170600	100170600	A	T
PLAU	nonsynonymous SNV	NM_001145031:c.655A>G:p.I219V	rs150389556	chr10	75673763	75673763	A	G
PLCB4	nonsynonymous SNV	NM_000933:c.1364A>G:p.K455R	rs142617224	chr20	9374275	9374275	A	G
SEMA4A	nonsynonymous SNV	NM_001193300:c.232G>A:p.V78M	n\a	chr1	156126297	156126297	G	A
SLC16A9	nonsynonymous SNV	NM_194298:c.1006G>T:p.V336L	n\a	chr10	61413778	61413778	C	A
UBOX5	nonsynonymous SNV	NM_199415:c.1427C>T:p.P476L	rs201389796	chr20	3090789	3090789	G	A

Criteria: nonsynonymous SNV; exons and splice sites affected; indel, stop loss or stop gain; average heterozygosity <0.01 or unknown; not seen in in-house exome data. The chromosomal positions refer to hg19. Because of its well known function, PLAU (plasminogen activator, urokinase) was not further analyzed. n\a, not available.

Supplementary Table 2 | All shared heterozygous variations of individuals K3, K13 and K18.

Gene	ExonicFunc	AAChange	dbSNP141	Chr	Start	End	Ref	Obs
ADAMTSL4	nonsynonymous SNV	NM_019032:c.3179G>A:p.R1060H	rs147697821	chr1	150532626	150532626	G	A
ANKRD28	nonsynonymous SNV	NM_001195099:c.1399A>G:p.I467V	n\a	chr3	15731538	15731538	T	C
ARSH	nonsynonymous SNV	NM_001011719:c.865T>G:p.Y289D	rs200903295	chrX	2936675	2936675	T	G
B3GALTL	nonsynonymous SNV	NM_194318:c.1325A>G:p.H442R	n\a	chr13	31898028	31898028	A	G
CDC25B	nonsynonymous SNV	NM_004358:c.400C>T:p.R134C	rs147172963	chr20	3781128	3781128	C	T
DCBLD2	nonsynonymous SNV	NM_080927:c.1247T>C:p.V416A	n\a	chr3	98531292	98531292	A	G
EPHA4	nonsynonymous SNV	NM_004438:c.2918G>A:p.R973Q	rs140023331	chr2	222290791	222290791	C	T
FILIP1	nonsynonymous SNV	NM_015687:c.1760C>T:p.S587F	n\a	chr6	76023788	76023788	G	A
FILIP1	nonsynonymous SNV	NM_015687:c.3356G>A:p.R1119Q	rs147434989	chr6	76022192	76022192	C	T
GBP5	nonsynonymous SNV	NM_001134486:c.1031C>T:p.P344L	n\a	chr1	89730487	89730487	G	A
MOGAT1	nonsynonymous SNV	NM_058165:c.655G>A:p.A219T	rs201526036	chr2	223559809	223559809	G	A
P2RY4	nonsynonymous SNV	NM_002565:c.674G>C:p.R225P	rs202027224	chrX	69478801	69478801	C	G
PODNL1	nonsynonymous SNV	NM_001146255:c.904C>T:p.R302C	n\a	chr19	14043880	14043880	G	A
PRR14L	nonsynonymous SNV	NM_173566:c.5956G>A:p.A1986T	n\a	chr22	32099580	32099580	C	T
RABGAP1L	nonsynonymous SNV	NM_014857:c.1706C>T:p.T569I	n\a	chr1	174363279	174363279	C	T
RTEL1	nonsynonymous SNV	NM_016434:c.2828C>T:p.P943L	n\a	chr20	62324333	62324333	C	T
SEMA4A	nonsynonymous SNV	NM_001193300:c.232G>A:p.V78M	n\a	chr1	156126297	156126297	G	A
SKA3	nonsynonymous SNV	NM_145061:c.1120C>T:p.L374F	n\a	chr13	21729950	21729950	G	A
SLC16A9	nonsynonymous SNV	NM_194298:c.1006G>T:p.V336L	n\a	chr10	61413778	61413778	C	A
SRSF12	nonframeshift deletion	NM_080743:c.356_358del:p.R119del	n\a	chr6	89814896	89814898	TTC	0
STT3B	nonsynonymous SNV	NM_178862:c.1150T>G:p.F384V	rs199778452	chr3	31659458	31659458	T	G
UBOX5	nonsynonymous SNV	NM_199415:c.1427C>T:p.P476L	rs201389796	chr20	3090789	3090789	G	A
ZNF660	nonsynonymous SNV	NM_173658:c.599G>A:p.G200D	rs150885666	chr3	44636284	44636284	G	A
ZNF763	nonsynonymous SNV	NM_001012753:c.634G>T:p.V212F	rs7249379	chr19	12089364	12089364	G	T

Criteria: nonsynonymous SNV; exons and splice sites affected; indel, stop loss or stop gain; average heterozygosity <0.001 or unknown; exclusion of low quality reads; not seen in in-house exome data. The chromosomal positions refer to hg19. n\a, not available.

Supplementary Table 3 | Patients' characteristics and *SEMA4A* screening results.

UPN	Sex	Neoplasm	Age at diagnosis (years)	Amsterdam	*SEMA4A* variants
MUG1	M	CRC	28	Modified	g.31812C>T, c.2044C>T, **p.Pro682Ser**, het. (rs76381440)
MUG2	M	PaC	47	I+II	g.31484C>T, c.1716C>T, p.Pro572=, hom. (rs12401573)
MUG3	M	CRC	13	I+II	g.31484C>T, c.1716C>T, p.Pro572=, het. (rs12401573)
BN01	F	CRC	43	I	g.31484C>T, c.1716C>T, p.Pro572=, hom. (rs12401573); g.16569C>T, c.977C>T, **p.Ser326Phe**, hom.
BN02	F	CRC	30	I	g.31484C>T, c.1716C>T, p.Pro572=, hom. (rs12401573)
BN03	M	CRC	47	I	g.31484C>T, c.1716C>T, p.Pro572=, het. (rs12401573)
BN04	F	OvC	27	I	g.30159G>C, c.1451G>C, **p.Gly484Ala**, het. (rs148744804)
BN05	F	CRC	40	I	g.31484C>T, c.1716C>T, p.Pro572=, hom. (rs12401573)
BN06	F	CRC	64	I	-
BN07	F	CRA	45	I	-
BN08	M	CRC	53	I	g.31484C>T, c.1716C>T, p.Pro572=, hom. (rs12401573)
BN09	M	CRC	53	I	g.31955G>A, c.2187G>A, p.Pro729=, het. (rs41265019)
BN10	F	CRC	75	I	g.31484C>T, c.1716C>T, p.Pro572=, het. (rs12401573)
BN11	M	CRC	61	I	g.31484C>T, c.1716C>T, p.Pro572=, het. (rs12401573); g.31812C>T, c.2044C>T, **p.Pro682Ser**, het. (rs76381440)
BN12	F	CRC	76	I	g.31484C>T, c.1716C>T, p.Pro572=, het. (rs12401573)
BN13	F	CRC	46	I	g.31484C>T, c.1716C>T, p.Pro572=, het. (rs12401573)
BN14	M	CRC	49	I	-
BN15	F	CRC	68	I	-
BN16	F	CRC	82	I	g.31484C>T, c.1716C>T, p.Pro572=, hom. (rs12401573)
BN17	F	CRA	64	I	g.31484C>T, c.1716C>T, p.Pro572=, het. (rs12401573)
BN18	M	CRC	36	I	g.31484C>T, c.1716C>T, p.Pro572=, hom. (rs12401573)
BN19	F	CRC	42	I	g.31484C>T, c.1716C>T, p.Pro572=, hom. (rs12401573)
BN20	F	CRC	40	I	g.31484C>T, c.1716C>T, p.Pro572=, het. (rs12401573)
BN21	M	CRC	52	I	g.31484C>T, c.1716C>T, p.Pro572=, hom. (rs12401573)
BN22	M	CRC	45	I	g.31484C>T, c.1716C>T, p.Pro572=, het. (rs12401573); g.31812C>T, c.2044C>T, **p.Pro682Ser**, het. (rs76381440)
BN23	F	CRC	45	I	g.31484C>T, c.1716C>T, p.Pro572=, hom. (rs12401573)
BN24	F	CRC	49	I	g.31484C>T, c.1716C>T, p.Pro572=, hom. (rs12401573)
BN25	F	CRC	43	I	-
BN26	M	CRC	48	I	g.31484C>T, c.1716C>T, p.Pro572=, het. (rs12401573); g.31812C>T, c.2044C>T, **p.Pro682Ser**, het. (rs76381440)
BN27	F	CRC	54	I	-
BN28	F	CRC	66	I	g.31484C>T, c.1716C>T, p.Pro572=, het. (rs12401573)
BN29	F	EnC	62	II	g.31484C>T, c.1716C>T, p.Pro572=, hom. (rs12401573)
BN30	M	CRC	45	I	g.31484C>T, c.1716C>T, p.Pro572=, hom. (rs12401573)
BN31	F	CRC	51	I	g.31484C>T, c.1716C>T, p.Pro572=, het. (rs12401573)
BN32	F	DCIS, CRC	38, 40	I	g.31484C>T, c.1716C>T, p.Pro572=, het. (rs12401573); g.31812C>T, c.2044C>T, **p.Pro682Ser**, het. (rs76381440)
BN33	M	CRA	48	I	g.31484C>T, c.1716C>T, p.Pro572=, hom. (rs12401573)
BN34	F	CRC	40	I	g.31484C>T, c.1716C>T, p.Pro572=, het. (rs12401573)
BN35	M	CRC	86	I	g.31484C>T, c.1716C>T, p.Pro572=, hom. (rs12401573)
BN36	M	CRC	47	I	g.31484C>T, c.1716C>T, p.Pro572=, het. (rs12401573)
BN37	F	CRC	56	I	g.31484C>T, c.1716C>T, p.Pro572=, het. (rs12401573)
BN38	M	CRC	49	I	g.31484C>T, c.1716C>T, p.Pro572=, het. (rs12401573)
BN39	F	CRC	50	II	-
BN40	M	CRC	60	I	g.31906G>A, c.2138G>A, p.Arg713Gln, het. (rs41265017)
BN41	M	CRC	48	I	g.31484C>T, c.1716C>T, p.Pro572=, het. (rs12401573)
BN42	M	CRC	51	I	g.31484C>T, c.1716C>T, p.Pro572=, het. (rs12401573); g.31812C>T, c.2044C>T, **p.Pro682Ser**, het. (rs76381440)
BN43	M	CRC	42	II	g.31484C>T, c.1716C>T, p.Pro572=, hom. (rs12401573)
BN44	M	CRC	44	II	g.31484C>T, c.1716C>T, p.Pro572=, het. (rs12401573)
BC17	F	CRC	56	I+II	g.31484C>T, c.1716C>T, p.Pro572=, hom. (rs12401573)
BC20	F	CRC	47	II	-
BC41	F	CRC	47	I	g.31484C>T, c.1716C>T, p.Pro572=, hom. (rs12401573)
BC95	F	CRC	47	I+II	g.31484C>T, c.1716C>T, p.Pro572=, hom. (rs12401573)
BC663	M	CRC	47	I	g.31484C>T, c.1716C>T, p.Pro572=, het. (rs12401573)
BC676	M	CRC	59	I	-

Variant coding refers to NG_027683.1, NM_001193300.1 and NP_001180229.1, respectively. CRA, colorectal adenoma; DCIS, ductal carcinoma in situ; EnC, endometrial cancer; PaC, pancreatic cancer; OvC, overian cancer.

Supplementary Table 4 | Mutation predictions of germline *SEMA4A* mutations.

Transcript	Protein	MutationTaster 2 probability value	MutationTaster 2	PP2 HumDiv Score	PP2 HumDiv	PP2 HumVar Score	PP2 HumVar	SIFT Score	SIFT	phyloP100wayall	phastCons100way	phastConsElements100way	GERP (position-specific score)
c.232G>A	p.Val78Met	0.95	Disease causing	1	Probably damaging	0.987	Probably damaging	0	Damaging	7.434	1	lod=65, 407	2.94
c.977C>T	p.Ser326Phe	1	Disease causing	0.995	Probably damaging	0.945	Probably damaging	0.01	Damaging	5.577	1	lod=27, 320	4.45
c.1451G>C	p.Gly484Ala	1	Disease causing	0.876	Possibly damaging	0.541	Possibly damaging	0.19	Tolerated	4.124	1	lod=59, 397	4.27

Supplementary Table 5 | Validated somatic *SEMA4A* amino acid altering variants in human cancers.

Tissue	Sample ID	Type	Chr	from	to	Reference	Change	dbSNP141	Transcript	Protein	MutationTaster	PP2 HumDiv	PP2 HumVar	SIFT (cutoff=0.05)	MSI status	APC	BRAF	PIK3CA	KRAS	TP53	Anatomic site	Histologic type
Bladder	TCGA-G2-A2EO-01A-11D-A17V-08	subs	1	156126258	156126258	G	C	n\a	NM_001193300.1:c.193G>C	NP_001180229.1:p.Asp65His	Disease causing	Probably damaging	Probably damaging	Damaging								
Bladder	TCGA-DK-A1A3-01A-11D-A13W-08	subs	1	156127890	156127890	G	C	n\a	NM_001193300.1:c.330G>C	NP_001180229.1:p.Lys110Asn	Polymorphism	Probably damaging	Probably damaging	Damaging								
Bladder	TCGA-GC-A3OO-01A-11D-A22Z-08	subs	1	156145022	156145022	C	G	n\a	NM_001193300.1:c.1580C>G	NP_001180229.1:p.Ser527Cys	Polymorphism	Probably damaging	Possibly damaging	Tolerated								
Breast	TCGA-AN-A0AT-01A-11D-A045-09	subs	1	156124481	156124481	C	T	n\a	NM_001193300.1:c.112C>T	NP_001180229.1:p.Pro38Ser	Polymorphism	Probably damaging	Probably damaging	Tolerated								
Breast	SA075	subs	1	156128533	156128533	G	T	n\a	NM_001193300.1:c.486G>T	NP_001180229.1:p.Leu162Phe	Polymorphism	Possibly damaging	Benign	Tolerated								
Breast	PD4980a	subs	1	156128548	156128548	C	A	n\a	NM_001193300.1:c.501C>A	NP_001180229.1:p.Asp167Glu	Polymorphism	benign	Benign	Tolerated								
Breast	TCGA-C8-A274-01A-11D-A16D-09	subs	1	156142776	156142776	C	G	n\a	NM_001193300.1:c.1294C>G	NP_001180229.1:p.Leu432Val	Polymorphism	benign	Benign	Tolerated								
Breast	TCGA-C8-A274-01A-11D-A16D-09	subs	1	156146369	156146369	C	T	n\a	NM_001193300.1:c.1867C>T	NP_001180229.1:p.Gln623*	Disease causing	n\a	n\a	n\a								
Breast	TCGA-AN-A0FK-01A-11W-A050-09	indel	1	156146568	156146573	TCCTCT	-	n\a	NM_001193300.1:c.2067_2072del	NP_001180229.1:p.Leu690_Phe691del	Disease causing	n\a	n\a	n\a								
Cervix	TCGA-DR-A0ZM-01A-12D-A10S-08	subs	1	156131286	156131286	C	G	n\a	NM_001193300.1:c.960C>G	NP_001180229.1:p.Ile320Met	Disease causing	Possibly damaging	Possibly damaging	Damaging								
Cervix	TCGA-FU-A23L-01A-11D-A16O-08	subs	1	156132829	156132829	G	A	n\a	NM_001193300.1:c.1078G>A	NP_001180229.1:p.Glu360Lys	Disease causing	Probably damaging	Probably damaging	Damaging								
Colorectum	TCGA-AZ-6608-01A-11D-1835-10	subs	1	156126225	156126225	A	T	n\a	NM_001193300.1:c.160A>T	NP_001180229.1:p.Ser54Cys	Polymorphism	Possibly damaging	Possibly damaging	Damaging	MSI-L	R186*, Arg1331*	No	No	E62G	P153Afs*28	n\a	Colon Adenocarcinoma
Colorectum	TCGA-AA-A00K-01A-02W-A005-10	indel	1	156126289	156126290	CT	-	n\a	NM_001193300.1:c.224_225delCT	NP_001180229.1:p.Tyr77Argfs*30	Disease causing	n\a	n\a	n\a	MSI-L	Q1349*	No	No	No	No	n\a	Colon Adenocarcinoma
Colorectum	TCGA-DM-A1HB-01A-21D-A183-10	subs	1	156127898	156127898	G	A	n\a	NM_001193300.1:c.338G>A	NP_001180229.1:p.Cys113Tyr	Disease causing	Probably damaging	Probably damaging	Damaging	MSI-H	R499L, S1272*, P2540S	No	No	No	I255T, V218A	Ascending Colon	Colon Mucinous Adenocarcinoma
Colorectum	TCGA-CK-5913-01A-11D-1650-10	subs	1	156130720	156130720	T	C	n\a	NM_001193300.1:c.710T>C	NP_001180229.1:p.Ile237Thr	Disease causing	Benign	Benign	Damaging	MSI-H	S940L, D1425G	L475R	No	No	No	Sigmoid Colon	Colon Adenocarcinoma
Colorectum	TCGA-AY-6197-01A-11D-1719-10	subs	1	156130764	156130764	A	G	n\a	NM_001193300.1:c.754A>G	NP_001180229.1:p.Ser252Gly	Polymorphism	Possibly damaging	Benign	Tolerated	MSI-H	S1501Ffs*13, R564*, T1705P	No	K986E	No	R175H	Sigmoid Colon	Colon Adenocarcinoma
Colorectum	TCGA-CM-5861-01A-01D-1650-10	subs	1	156131159	156131159	T	G	n\a	NM_001193300.1:c.833T>G	NP_001180229.1:p.Leu278Arg	Disease causing	Probably damaging	Probably damaging	Damaging	MSI-H	M44V	No	K179R, E365G	No	No	Ascending Colon	Colon Adenocarcinoma
Colorectum	TCGA-CK-5915-01A-11D-1650-10	subs	1	156131242	156131242	C	T	n\a	NM_001193300.1:c.916C>T	NP_001180229.1:p.His306Tyr	Disease causing	Probably damaging	Probably damaging	Damaging	MSS	S1202P, C1387R, Q1541*, E1552G	No	No	No	No	Cecum	Colon Adenocarcinoma
Colorectum	TCGA-AA-3950-01A-02W-0995-10	subs	1	156142624	156142624	T	C	n\a	NM_001193300.1:c.1142T>C	NP_001180229.1:p.Val381Ala	Polymorphism	Benign	Benign	Tolerated	MSI-H	S1465Wfs*3	G615R	S405Y, E522V, G1050S	No	No	Sigmoid Colon	Colon Adenocarcinoma
Colorectum	TCGA-D5-6928-01A-11D-1924-10	subs	1	156142667	156142667	T	A	n\a	NM_001193300.1:c.1185T>A	NP_001180229.1:p.His395Gln	Disease causing	Probably damaging	Probably damaging	Damaging	MSI-H	S1315P	No	No	No	No	Cecum	Colon Adenocarcinoma
Colorectum	TCGA-AD-6889-01A-11D-1924-10	subs	1	156142773	156142773	C	A	n\a	NM_001193300.1:c.1291C>A	NP_001180229.1:p.His431Asn	Polymorphism	Possibly damaging	Benign	Damaging	MSI-H	No	No	No	T20A	No	Sigmoid Colon	Colon Adenocarcinoma
Colorectum	587232 (doi:10.1038/nature11282)	subs	1	156144967	156144967	G	T	n\a	NM_001193300.1:c.1525G>T	NP_001180229.1:p.Ala509Ser	Disease causing	Probably damaging	Probably damaging	Tolerated	MSS	No	No	C604R	No	No	Cecum	Colon Adenocarcinoma
Colorectum	TCGA-CK-5916-01A-11D-1650-10	subs	1	156145439	156145439	C	T	n\a	NM_001193300.1:c.1685C>T	NP_001180229.1:p.Pro562Leu	Disease causing	Possibly damaging	Benign	Damaging	MSI-H	R380L, S1278P, S1495G	No	N1044S	No	No	Cecum	Colon Adenocarcinoma
Colorectum	587386 (doi:10.1038/nature11282)	subs	1	156146474	156146474	C	T	n\a	NM_001193300.1:c.1972C>T	NP_001180229.1:p.Arg658Trp	Disease causing	Probably damaging	Possibly damaging	Damaging	MSS	No	No	No	No	No	Ascending Colon	Colon Mucinous Adenocarcinoma
Colorectum	TCGA-DC-5337-01A-01D-1657-10	subs	1	156146478	156146478	A	G	n\a	NM_001193300.1:c.1976A>G	NP_001180229.1:p.Glu659Gly	Disease causing	Benign	Benign	Tolerated	n\a	No	No	No	No	No	n\a	n\a
Colorectum	TCGA-AA-3984-01A-02W-0995-10	subs	1	156146732	156146732	A	G	n\a	NM_001193300.1:c.2230A>G	NP_001180229.1:p.Thr744Ala	Polymorphism	Benign	Benign	Damaging	MSS	F2784C	No	No	No	No	Transverse Colon	Colon Mucinous Adenocarcinoma
Esophagus	ESO-173-Tumor	subs	1	156130752	156130752	G	A	n\a	NM_001193300.1:c.742G>A	NP_001180229.1:p.Glu248Lys	Polymorphism	Possibly damaging	benign	Damaging								
Esophagus	ESO-1488-Tumor	subs	1	156132838	156132838	C	T	n\a	NM_001193300.1:c.1087C>T	NP_001180229.1:p.Arg363Cys	Disease causing	Probably damaging	Probably damaging	Damaging								
Head&Neck	TCGA-CN-6013-01A-11D-1683-08	subs	1	156128225	156128225	C	T	n\a	NM_001193300.1:c.410C>T	NP_001180229.1:p.Thr137Ile	Disease causing	Probably damaging	Probably damaging	Damaging								
Head&Neck	TCGA-CV-7101-01A-11D-2012-08	subs	1	156142765	156142765	G	A	n\a	NM_001193300.1:c.1283G>A	NP_001180229.1:p.Gly428Glu	Disease causing	Probably damaging	Probably damaging	Damaging								
Head&Neck	TCGA-BB-4223-01A-01D-1434-08	subs	1	156142792	156142792	G	A	n\a	NM_001193300.1:c.1310G>A	NP_001180229.1:p.Gly437Glu	Disease causing	Probably damaging	Probably damaging	Damaging								
Lung Adeno	LUAD-F00365-Tumor	subs	1	156126304	156126304	C	T	n\a	NM_001193300.1:c.239C>T	NP_001180229.1:p.Ala80Val	Disease causing	Possibly damaging	Possibly damaging	Damaging								
Lung Squamous	TCGA-34-5231-01A-21D-1817-08	subs	1	156144709	156144709	A	G	n\a	NM_001193300.1:c.1412A>G	NP_001180229.1:p.Asn471Ser	Disease causing	benign	Benign	Tolerated								
Lung Squamous	TCGA-18-3410-01A-01D-0983-08	subs	1	156146475	156146475	G	T	n\a	NM_001193300.1:c.1973G>T	NP_001180229.1:p.Arg658Leu	Disease causing	benign	Benign	Damaging								
Melanoma	TCGA-FS-A1ZQ-06A-11D-A197-08	subs	1	156130705	156130705	C	T	n\a	NM_001193300.1:c.695C>T	NP_001180229.1:p.Ser232Phe	Disease causing	Probably damaging	Possibly damaging	Damaging								
Melanoma	TCGA-EE-A29L-06A-12D-A196-08	subs	1	156132739	156132739	G	A	n\a	NM_001193300.1:c.988G>A	NP_001180229.1:p.Val330Ile	Polymorphism	benign	Benign	Tolerated								
Melanoma	SKCM-Ma-Mel-114-Tumor	subs	1	156132836	156132836	C	T	n\a	NM_001193300.1:c.1085C>T	NP_001180229.1:p.Ser362Leu	Disease causing	benign	Benign	Tolerated								
Melanoma	TCGA-EE-A2MF-06A-11D-A21A-08	subs	1	156146255	156146255	G	A	n\a	NM_001193300.1:c.1753G>A	NP_001180229.1:p.Ala585Thr	Disease causing	benign	Benign	Tolerated								
Melanoma	TCGA-IH-A3EA-01A-11D-A20D-08	subs	1	156146328	156146328	C	T	n\a	NM_001193300.1:c.1826C>T	NP_001180229.1:p.Ser609Phe	Disease causing	Probably damaging	Probably damaging	Damaging								
Melanoma	TCGA-EE-A2MR-06A-11D-A196-08	subs	1	156146351	156146351	G	A	n\a	NM_001193300.1:c.1849G>A	NP_001180229.1:p.Gly617Arg	Polymorphism	Possibly damaging	Benign	Tolerated								
Melanoma	SKCM-Ma-Mel-94-Tumor	subs	1	156146391	156146391	G	A	n\a	NM_001193300.1:c.1889G>A	NP_001180229.1:p.Gly630Asp	Disease causing	benign	Benign	Tolerated								
Melanoma	ME037-Tumor	subs	1	156146412	156146412	C	T	n\a	NM_001193300.1:c.1910C>T	NP_001180229.1:p.Ser637Phe	Polymorphism	Probably damaging	Probably damaging	Tolerated								
Melanoma	PD9028a	subs	1	156146606	156146606	C	T	n\a	NM_001193300.1:c.2104C>T	NP_001180229.1:p.Leu702Phe	Polymorphism	benign	Benign	Tolerated								
Melanoma	TCGA-EE-A29C-06A-21D-A197-08	subs	1	156146619	156146619	C	T	n\a	NM_001193300.1:c.2117C>T	NP_001180229.1:p.Pro706Leu	Disease causing	benign	Benign	Tolerated								
Ovary	TCGA-36-1568-01A-01W-0615-10	subs	1	156131281	156131281	C	T	n\a	NM_001193300.1:c.955C>T	NP_001180229.1:p.His319Tyr	Polymorphism	benign	Benign	Tolerated								
Prostate	PR-1024-Tumor	subs	1	156130795	156130795	C	T	n\a	NM_001193300.1:c.785C>T	NP_001180229.1:p.Thr262Ile	Polymorphism	benign	Benign	Tolerated								
Prostate	TCGA-CH-5771-01A-21D-1576-08	subs	1	156131152	156131152	G	A	n\a	NM_001193300.1:c.826G>A	NP_001180229.1:p.Glu276Lys	Disease causing	Possibly damaging	Possibly damaging	Damaging								
Prostate	PR-00-1823-Tumor	subs	1	156132872	156132872	C	G	n\a	NM_001193300.1:c.1121C>G	NP_001180229.1:p.Pro374Arg	Disease causing	Probably damaging	Probably damaging	Damaging								
Stomach	TCGA-CD-5813-01A-11D-1600-08	subs	1	156128511	156128511	A	T	n\a	NM_001193300.1:c.464A>T	NP_001180229.1:p.Glu155Val	Disease causing	Probably damaging	Possibly damaging	Tolerated								
Stomach	TCGA-BR-4298-01A-01D-1126-08	subs	1	156130789	156130789	T	C	n\a	NM_001193300.1:c.779T>C	NP_001180229.1:p.Leu260Pro	Disease causing	Probably damaging	Possibly damaging	Damaging								
Stomach	TCGA-BR-7703-01A-11D-2053-08	subs	1	156142686	156142686	G	C	n\a	NM_001193300.1:c.1204G>C	NP_001180229.1:p.Val402Leu	Disease causing	Probably damaging	Probably damaging	Damaging								
Stomach	TCGA-BR-4200-01A-01D-1126-08	subs	1	156144956	156144956	A	G	n\a	NM_001193300.1:c.1514A>G	NP_001180229.1:p.Asp505Gly	Disease causing	Probably damaging	Possibly damaging	Damaging								
Stomach	TCGA-CG-4305-01A-01D-1158-08	subs	1	156146488	156146488	G	T	n\a	NM_001193300.1:c.1986G>T	NP_001180229.1:p.Lys662Asn	Polymorphism	benign	Benign	Tolerated								
Stomach	TCGA-BR-4256-01A-01D-1126-08	subs	1	156146693	156146693	A	G	n\a	NM_001193300.1:c.2191A>G	NP_001180229.1:p.Ser731Gly	Disease causing	Possibly damaging	Possibly damaging	Tolerated								
Uterus	TCGA-AP-A059-01A-21D-A122-09	subs	1	156124433	156124433	C	A	n\a	NM_001193300.1:c.64C>A	NP_001180229.1:p.Leu22Ile	Polymorphism	benign	Benign	Tolerated								
Uterus	TCGA-AP-A0LT-01A-11W-A062-09	subs	1	156131239	156131239	C	T	n\a	NM_001193300.1:c.913C>T	NP_001180229.1:p.Arg305Cys	Disease causing	Probably damaging	Probably damaging	Damaging								
Uterus	TCGA-D1-A163-01A-11D-A12J-09	subs	1	156132838	156132838	C	T	n\a	NM_001193300.1:c.1087C>T	NP_001180229.1:p.Arg363Cys	Disease causing	Probably damaging	Probably damaging	Damaging								
Uterus	TCGA-D1-A17B-01A-22D-A12J-09	subs	1	156132886	156132886	G	T	n\a	NM_001193300.1:c.1134+1G>T	NP_001180229.1:p.?	Disease causing	n\a	n\a	n\a								
Uterus	TCGA-AP-A054-01A-11W-A062-09	subs	1	156142782	156142782	A	G	n\a	NM_001193300.1:c.1300A>G	NP_001180229.1:p.Met434Val	Disease causing	benign	Benign	Damaging								
Uterus	TCGA-BS-A0UV-01A-11D-A10B-09	subs	1	156144705	156144705	C	T	n\a	NM_001193300.1:c.1408C>T	NP_001180229.1:p.Arg470Cys	Polymorphism	Probably damaging	Possibly damaging	Damaging								
Uterus	TCGA-AX-A0IS-01A-12D-A10M-09	indel	1	156144922	156144923	-	G	n\a	NM_001193300.1:c.1481dupG	NP_001180229.1:p.Ala495Serfs*4	Disease causing	n\a	n\a	n\a								
Uterus	TCGA-AP-A0LE-01A-11D-A127-09	subs	1	156145423	156145423	C	T	n\a	NM_001193300.1:c.1669C>T	NP_001180229.1:p.Arg557Trp	Polymorphism	Probably damaging	Possibly damaging	Damaging								

Variants could be identified in 2.2% (3/136) of Bladder, 0.6% (6/963) of Breast, 5.3% (2/38) of Cervical, 2.7% (15/559) of Colorectal, 1.4% (2/146) of Esophageal, 0.8% (3/380) of H&N, 1.1% (2/176) of Lung Squamous, 2.5% (10/396) of Melanoma, 0.2% (1/471) of Ovary, 0.9% (3/330) of Prostate, 2.8% (6/212) of Stomach and 3.3% (8/241) Uterine Cancers, respectively. 92% of variants are missense. Additional information was extracted for CRCs. Data refers to Nature. 2013 Aug 22;500(7463):415-21. doi: 10.1038/nature12477. n\a, not available.

Supplementary Table 6 | Primers used in the screening of the 14 coding regions of *SEMA4A*.

Oligoname	Sequence (5' to 3')	Amplicon size (bp) w\o M13 tag sequence
SEMA4A_Ex3_fw	tgtaaaacgacggccagAATACACACGCTTCTGCTGC	347
SEMA4A_Ex3_rv	caggaaacagctatgaccCCTCTGTTCCTCTCTCCTTCC	
SEMA4A_Ex4_fw	tgtaaaacgacggccagGGAGCACACTCAGGCAACC	347
SEMA4A_Ex4_rv	caggaaacagctatgaccCATGCACAGGCAGCCAAG	
SEMA4A_Ex5_fw	tgtaaaacgacggccagCACTAACCACCATGTCTGCTG	305
SEMA4A_Ex5_rv	caggaaacagctatgaccAATCCAGGAGATAACCGTGC	
SEMA4A_Ex6_fw	tgtaaaacgacggccagCTCTGGGGGTCCAGCAATTT	268
SEMA4A_Ex6_rv	caggaaacagctatgaccGTCCTGCATCTGGAAGGC	
SEMA4A_Ex7_fw	tgtaaaacgacggccagGATGTGAGACCTTGGCGTTC	273
SEMA4A_Ex7_rv	caggaaacagctatgaccATGCACGCATCACCACAC	
SEMA4A_Ex8_fw	tgtaaaacgacggccagAGAGGCAGGTCTGTGGAGG	281
SEMA4A_Ex8_rv	caggaaacagctatgaccGGACACATTGTACCTTCCGC	
SEMA4A_Ex9_fw	tgtaaaacgacggccagGAGGAAGCCTGTGTGTCCTG	313
SEMA4A_Ex9_rv	caggaaacagctatgaccCCACCACTCAGTCCTGCC	
SEMA4A_Ex10_ext_fw	GGCCAAACCAACGGTTTTC	1029
SEMA4A_Ex10_ext_rv	TTGGCCTTACCCTTGGCTTCT	
SEMA4A_Ex10_in_fw	TTTGTGGCAGCCATCCCTT	840
SEMA4A_Ex10_in_rv	TCCCTTTTGGAACTAGGCAGAA	
SEMA4A_Ex11_fw	tgtaaaacgacggccagAGAGAGAGCTGCTGGTGTGG	335
SEMA4A_Ex11_rv	caggaaacagctatgaccGAGCAGGCGAGATTGGTG	
SEMA4A_Ex12_fw	tgtaaaacgacggccagCAACCTGATCTGCCTCCCTC	261
SEMA4A_Ex12_rv	caggaaacagctatgaccGGCATCCTCTGCTCTAGTCC	
SEMA4A_Ex13-14_fw	tgtaaaacgacggccagTTTCTTACAGCTGGGGAGGC	574
SEMA4A_Ex13-14_rv	caggaaacagctatgaccAGCAGAAGCTGGCTGAGAAG	
SEMA4A_Ex15_fw	tgtaaaacgacggccagCCTGGCCTACCTTCTTCC	316
SEMA4A_Ex15_rv	caggaaacagctatgaccCAGATCTCAAGCCAGGCAG	
SEMA4A_Ex16-1_fw	tgtaaaacgacggccagGGCTGGGGTCCAAAGATAGG	496
SEMA4A_Ex16-1_rv	caggaaacagctatgaccATGAGGGCTCCTGAAAGCAC	
SEMA4A_Ex16-2_fw	tgtaaaacgacggccagGATCCTGAACTGGCAGGCAT	n\a
SEMA4A_Ex16-2_rv	caggaaacagctatgaccCATCCTAGTCAGGGCTGTGC	

Due to low complexity regions and high GC-content, sequencing of exon 10 could only be accomplished by a nested PCR approach without M13 sequence tagging.
